# Medical Management and Prevention of Motor Complications in Parkinson’s Disease

**DOI:** 10.1007/s13311-020-00889-4

**Published:** 2020-08-05

**Authors:** Stephen D. Aradi, Robert A. Hauser

**Affiliations:** Department of Neurology, Parkinson’s Foundation Center of Excellence, University of South Florida, Tampa, FL USA

**Keywords:** Parkinson’s disease, treatment, levodopa, motor fluctuations, levodopa-induced dyskinesia, continuous dopaminergic stimulation

## Abstract

Levodopa is the most effective medication for the treatment of the motor symptoms of Parkinson’s disease. However, over time, the clinical response to levodopa becomes complicated by a reduction in the duration and reliability of motor improvement (motor fluctuations) and the emergence of involuntary movements (levodopa-induced dyskinesia). Strategies that have been attempted in an effort to delay the development of these motor complications include levodopa sparing and continuous dopaminergic therapy. Once motor complications occur, a wide array of medical treatments is available to maximize motor function through the day while limiting dyskinesia. Here, we review the clinical features, epidemiology, and risk factors for the development of motor complications, as well as strategies for their prevention and medical management.

**Electronic supplementary material:**

The online version of this article (10.1007/s13311-020-00889-4) contains supplementary material, which is available to authorized users.

## Introduction

The cardinal motor features of Parkinson’s disease (PD) are bradykinesia (slowness characterized by decreased velocity and amplitude of repetitive movements), rigidity (velocity-independent increased resistance to passive movement about a joint), and tremor (characteristically a resting tremor of 4–6 Hz frequency) [1]. These features, with the variable exception of tremor, are typically improved by treatment with levodopa, and in early disease, this beneficial response is well-maintained by intermittent dosing during waking hours. Over time, however, the duration and reliability of symptomatic benefit declines, leading to waking time being divided into time during which levodopa is providing good benefit, when responsive parkinsonian signs and symptoms are reduced and functional status is improved (ON), and time during which levodopa is not providing good benefit, when parkinsonian signs or symptoms re-emerge and functional status declines (OFF) [2–4]. Transitions between ON and OFF states are referred to as motor fluctuations [2, 3].

There are several characteristic types of OFF. Gradual deterioration in the symptomatic response to a dose of levodopa prior to the next dose is commonly termed “wearing off” or “end-of-dose deterioration.” In more advanced disease, the duration of the levodopa response can be significantly shortened and patients may experience very rapid transitions between ON and OFF, termed “ON-OFF fluctuations.” Some patients can unexpectedly and suddenly transition from an ON state to an OFF state, unrelated to the timing of levodopa intake, and these OFF states are termed “sudden” or “random” OFFs. A “delayed ON” is said to occur when the time-to-ON following a levodopa dose is longer than usual, whereas a “dose failure” occurs when there is no benefit following a dose. A “partial ON” occurs when the benefit from a dose of levodopa is less robust than is typical for a given patient. The presence of OFF symptoms in the morning, prior to the first dose of levodopa, is commonly referred to as “early morning akinesia” or “early morning OFF” and is likely related to loss of benefit from the previous day’s medication [2, 5]. For evaluations in clinical trials, patients may be seen in the so-called practically defined OFF state, in the morning after not having taken Parkinson’s disease medications since the evening before, at least 12 h previously. Patients may also experience fluctuations in relation to other PD medications, especially apomorphine (as it also has robust efficacy) and less commonly in relation to other dopamine agonists [6].

In addition to fluctuations in motor symptoms, patients may also experience fluctuations in nonmotor symptoms including sensory, neuropsychiatric, and autonomic symptoms. To qualify as a nonmotor fluctuation, there should be evidence that the nonmotor symptom fluctuates in relation to the patient’s dopaminergic state, for example, emerging in association with a motor OFF state or improving following administration of levodopa (or other dopaminergic medications) [2].

In addition to the emergence of motor fluctuations, levodopa therapy may become complicated by involuntary movements, termed levodopa-induced dyskinesia (LID). LID comprise a variety of phenomenologies with different relationships to phases of the levodopa dosing cycle. The most common form of LID consists of hyperkinetic movements that occur during the peak of the levodopa response (peak-dose dyskinesia). These typically manifest as chorea, ballism, stereotypy, and less commonly dystonia or myoclonus; in many patients, peak-dose dyskinesia is more severe ipsilateral to the side with more severe parkinsonism and OFF period dystonia [3, 7]. Dyskinesia may also occur immediately following and preceding levodopa doses as plasma levodopa levels are rising and falling. These dyskinesia predominantly consist of dystonia, or more rarely ballism, often involve the lower extremities, and are referred to as “diphasic dyskinesia” or “dystonia-improvement-dystonia” (DID) [8]. Other rarer behaviors that have been described in relation to levodopa dosing include abnormal involuntary eye movements (often stereotyped upward or horizontal gaze deviations that can be jerky, tonic, or more sustained), disordered breathing, involuntary abdominal movements (“bellydancer’s dyskinesia”), and compulsive handling, sorting, or arranging of objects (“punding”) [9–17]. During OFF episodes, and especially during early morning akinesia, patients may develop painful dystonia, often involving the lower extremity with inversion and plantar flexion of the feet and toes, although dystonia of other body regions may occur as well. Dystonia involving the lower face that emerges early in the course of illness may suggest atypical parkinsonism, especially multiple systems atrophy, rather than PD [18, 19].

### Prevalence, Incidence, and Risk Factors for Motor Complications

Nearly all patients develop motor fluctuations and LID by 15 to 20 years from time of diagnosis [20, 21]. However, prevalence and incidence figures through the course of PD vary depending on the study methodology employed and by the predominant treatment strategies of the time. Early literature suggested that approximately 10% of patients per year following initiation of treatment with levodopa develop motor fluctuations, with 40% of patients developing these complications within 4–6 years of treatment [21, 22]. A large cross-sectional study of 617 patients with PD found an overall prevalence of wearing off of 57% as assessed by neurologists and 67% as assessed by a patient-completed questionnaire (19 item Wearing Off Questionnaire, WOQ-19) [23]. Of patients with disease duration < 2.5 years, wearing off was identified in 41.8% by the WOQ-19 and in 21.8% by neurologists, indicating that motor fluctuations can emerge as early as several months to a few years after the initiation of levodopa, as has also been observed in other studies [23, 24]. A retrospective analysis of an incident cohort of PD found estimated rates of dyskinesia of 30% by 5 treatment years and 59% by 10 treatment years [25].

A recent large prospective study followed an inception cohort of 734 patients with PD for up to 10 years from diagnosis [26]. Overall, 34.7% developed motor fluctuations and 25.3% developed dyskinesia during the course of the study. The conditional survival risk (chance of remaining free from an outcome) for developing motor fluctuations declined from 0.83 at < 3.5 years to 0.41 at 5–6.5 years whereas the conditional survival risk for developing dyskinesia declined from .87 at < 3.5 years to .59 at 5–6.5 years. Higher levodopa dose, favorable medication response, younger age at symptom onset, and greater nonmotor symptom burden were significantly associated with both motor fluctuations and dyskinesia. Higher education level was associated with motor fluctuations only and lower BMI was associated with dyskinesia only.

In an analysis of a clinical trial (CALM-PD) in which patients with early PD were randomized to blinded treatment with levodopa or pramipexole to which (additional) levodopa could be added as necessary, factors significantly associated with earlier occurrence of motor fluctuations were higher cumulative levodopa dose, higher cumulative levodopa equivalent dose, and occurrence of dyskinesia. Factors associated with later occurrence of motor fluctuations were age at onset ≥ 65 years and pramipexole treatment. Factors significantly associated with earlier occurrence of dyskinesia were more severe disease (Hoehn and Yahr stage ≥ 2), higher cumulative levodopa dose, higher cumulative levodopa equivalent dose, and occurrence of motor fluctuations. Pramipexole treatment was associated with a later occurrence of dyskinesia [27].

In an analysis of a study comparing initial treatment with levodopa/carbidopa *versus* levodopadopa/carbidopa/entacapone (STRIDE-PD), factors predictive of time to wearing off were lower age at onset, more severe disease (higher UPDRS Part II or Part III score), region (North America > Europe), higher levodopa dose, and female gender. Factors predictive of time to dyskinesia were lower age at onset, higher levodopa dose, region (North America > Europe), lower weight, treatment with levodopa/carbidopa/entacapone, female gender, and more severe disease (higher UPDRS Part II score) [28].

Thus, overall, the strongest predictors of motor complications appear to be younger age at onset, worse disease severity, and higher levodopa dose. Clinical observations and recent studies suggest that the prevalence of dyskinesia is lower today than it had been in previous eras, probably owing to clinicians’ tendency to employ lower levodopa doses through the course of the disease now than in the past [29–31].

### Prevention of Motor Complications

Strategies to delay or prevent the development of motor complications represent an unmet need in the treatment of PD. As yet, no therapies exist which have been proven to slow the progression of disease. Such disease-modifying therapies would clearly represent the most impactful interventions to prevent the development of motor complications, and the state of work on these efforts is described elsewhere. Other strategies that have been considered in an attempt to delay motor complications include utilizing existing symptomatic therapies as part of levodopa-sparing regimens and efforts to achieve continuous dopaminergic stimulation (CDS).

#### Choice of Initial Therapy: Levodopa-Sparing Therapies

Although an association between chronic levodopa use and higher levodopa doses and the risk of motor complications is well-established, how this information should influence clinical decision-making has been debated. One possible approach to decreasing the risk of motor complications is to employ a “levodopa-sparing” strategy by introducing non-levodopa PD medications first in early disease and adding levodopa only when symptoms are not adequately controlled by these other medications. Several clinical trials evaluated the strategy of initiating symptomatic therapy in early PD with levodopa *versus* a dopamine agonist to which (more) levodopa could be added as necessary [6, 32–35]. In general, these studies showed that introducing a dopamine agonist to which levodopa could be added lead to a lower incidence of motor fluctuations and dyskinesia for several years, but adverse events such as hallucinations, somnolence, and edema were more common in the dopamine agonist group and parkinsonian motor features were more improved in the levodopa group. Long-term disability was ultimately similar in both groups. Thus, no clear advantage was observed for using a dopamine agonist as initial therapy to spare levodopa. A pragmatic, randomized, open-label study in which patients with newly diagnosed PD were randomized to levodopa, a dopamine agonist, or a MAO-B inhibitor, found that patients treated with levodopa had better PDQ-39 mobility scores than those treated with a dopamine agonist or MAO-B inhibitor, and patients randomized to a levodopa-sparing approach discontinued those medications due to side effects at a higher rate than those treated with levodopa [36]. In light of these findings, and with the increased recognition of additional adverse effects of dopamine agonists such as impulse control disorders and sudden onset sleep, this strategy has mostly fallen out of favor, except perhaps in the very young patient.

Another possible strategy is to simply delay the introduction of levodopa as much as possible. In a delayed-start trial of levodopa in early PD, patients were assigned to levodopa for 80 weeks or placebo for 40 weeks followed by levodopa for 40 weeks. At 80 weeks, neither the rates of motor complications nor change from baseline in total Unified Parkinson Disease Rating Scale (UPDRS) scores were significantly different between groups [37]. Another study used a nested matched subgroups approach to compare patients from a large PD cohort in Ghana, in which access to medication is limited and the initiation of levodopa therapy often occurs many years after onset, to a group of patients in Italy who were recruited during the same period [38]. Although levodopa therapy was introduced later in Ghana (time from disease onset 4.2 *vs* 2.4 years, *p* < 0.001), disease duration at the occurrence of motor fluctuations and dyskinesia was similar in the two populations. Median disease duration at the first appearance of motor fluctuations and dyskinesia was comparable between Ghanaian and Italian patients (6.0 *vs* 5.5 years, *p =* 0.149 for motor fluctuations; 7.0 *vs* 6.5 years, *p =* 0.567 for dyskinesia), despite significantly shorter median duration of levodopa therapy at their onset in Ghanaians (0.5 *vs* 2.0 years, *p =* 0.001 for motor fluctuations; 1.0 *vs* 3.0 years, *p =* 0.004 for dyskinesia). In a multivariate analysis, disease duration and levodopa daily dose (mg/kg of body weight) were associated with motor complications, whereas the disease duration at the initiation of levodopa was not. Thus, in this study, onset of motor complications was strongly associated with disease duration and not time on levodopa [38].

Based on these observations, most experts introduce levodopa when the patient requires it for symptomatic and functional benefit as evidence is lacking to indicate that it should be started immediately upon diagnosis (irrespective of any functional impairment) or that it should be delayed as long as possible. However, there is general consensus, with good supportive evidence, that levodopa doses above those required for adequate control of symptoms should be avoided as higher doses may unnecessarily increase the risk for motor fluctuations and dyskinesia.

#### Continuous Dopaminergic Stimulation

Current evidence, although incomplete, suggests that motor fluctuations and dyskinesia are related to the short serum half-life of levodopa and the progressive depletion of nigrostriatal terminals, with resultant disordered handling of levodopa and release of dopamine (including by adjacent serotonergic terminals not optimized to store and release dopamine), and consequent synaptic and postsynaptic changes within the striatum. In early disease, sufficient numbers of nigrostriatal terminals (and compensatory mechanisms) are intact such that levodopa can be taken up and converted to dopamine, which is then stored and slowly released in a manner that maintains relatively continuous, physiologic dopaminergic tone and permits symptomatic benefit that lasts from dose to dose. As more dopamine neurons are lost, levodopa-derived dopamine storage and release is compromised, resulting in pulsatile postsynaptic stimulation, with dopamine peaks and troughs mirroring serum levodopa levels. Over time, the clinical response more and more closely mirrors fluctuations in levodopa serum concentrations [39–41].

These concepts have given rise to the idea of continuous dopaminergic stimulation (CDS) as a strategy to minimize motor fluctuations and dyskinesia. If therapeutic levels of levodopa were maintained through the day, one would expect motor fluctuations to be prevented or eliminated. Dyskinesia appear to be the result of a “sensitivity” that occurs as a result of fluctuating dopamine levels in the striatum. If physiologic dopamine levels were maintained in the striatum, the development of dyskinesia might be avoided. Indeed (as will be discussed in subsequent sections), infusion therapies consisting of continuous carbidopa/levodopa enteral suspension (CLES) or continuous subcutaneous apomorphine infusion (CSAI) significantly reduce established motor complications in patients with advanced PD [42–44].

Thus far, infusion therapies have not been studied in early PD to determine if they can reduce or eliminate the development of motor complications. The STRIDE-PD study, which randomized patients with early PD to levodopa/carbidopa (LC) alone or levodopa/carbidopa/entacapone (LCE) 4 times daily at 3.5-h intervals (in an effort to achieve CDS), unexpectedly found that patients receiving LCE experienced a shorter latency to developing dyskinesia. The investigators hypothesized that LCE does not sufficiently maintain levodopa concentrations to truly provide CDS and that the increase in dyskinesia was due to the higher levodopa equivalent doses provided by LCE compared to LC [45]. However, longer acting oral levodopa formulations such as carbidopa and levodopa extended release are now available and even longer acting ones (e.g., IPX203) are in development. These may be tested in future clinical trials. Alternative means to achieving CDS could theoretically include other highly effective, long duration, oral, transdermal, subcutaneous, depot, or infusion-delivered dopaminergic medications, as well as cell-based and gene therapy approaches [46].

## Medical Management of Motor Complications

Medication approaches to the management of motor fluctuations include shortening the interdose interval of carbidopa-levodopa immediate release (CD-LD IR), switching to a longer acting CD-LD oral formulation, adding a maintenance or on-demand adjunctive medication, or employing infusion therapy. Medication management of peak-dose LID may include reducing individual levodopa doses, fractionating the levodopa dose (administering smaller doses more frequently), or adding an amantadine formulation. In the following sections, evidence for these medication options is reviewed, followed by a discussion of the clinical application of these options during the course of advancing PD. These medications are also summarized in Table 1.

**Table 1:** Medications in the treatment of motor complications in Parkinson’s disease

Medication by class	FDA-approved indication	Clinical use^a^	Typical starting dose	Common and notable adverse effects^b^
Levodopa formulations				
Carbidopa/levodopa IR	Treatment of the symptoms of idiopathic Parkinson’s disease, postencephalitic parkinsonism, and symptomatic parkinsonism	Monotherapy in early disease; frequency and dose adjustments, and with adjunctive therapies in fluctuating disease	25 mg/100 mg three times daily, increase as needed	Dyskinesia, nausea, hallucinations, confusion, dizziness, orthostatic hypotension
Carbidopa/levodopa CR	Treatment of the symptoms of idiopathic PD, postencephalitic parkinsonism, and symptomatic parkinsonism	Monotherapy in early disease; in combination with adjunctive therapies in fluctuating disease; does not reduce OFF time *versus* IR CD-LD	50 mg/200 mg twice daily, increase as needed	Dyskinesia, nausea, hallucinations, confusion, dizziness, orthostatic hypotension
Carbidopa/levodopa ER	Treatment of the symptoms of idiopathic PD, postencephalitic parkinsonism, and symptomatic parkinsonism	Monotherapy in early disease; alone or with adjunctive therapies in fluctuating disease; reduces OFF time; increases ON time without troublesome dyskinesia	23.75 mg/95 mg three times daily in levodopa naïve; conversion from CD/LD IR per package insert; in clinical trials generally 2× IR daily dose divided 3, 4, or 5 times daily, with early feedback and adjustments	Nausea, dizziness, headache, insomnia, abnormal dreams, dry mouth, dyskinesia, anxiety, constipation, vomiting, orthostatic hypotension
Levodopa inhalation powder	Intermittent treatment of OFF episodes	As needed, intermittent treatment of OFF episodes	Two capsules (84 mg) up to 5 times daily	Cough, nausea, upper respiratory tract infection, and sputum discolored
Nonergoline dopamine receptor agonists				
Ropinirole	Treatment of signs and symptoms of Parkinson’s disease	Monotherapy in early disease; may delay development of motor complications; adjunct to levodopa in fluctuating disease; reduces OFF time; increases ON time without troublesome dyskinesia	IR: 0.25 mg three times daily PR: 2 mg daily Increase weekly to maximum 24 mg daily	Impulse control disorders, excessive and sudden daytime sleepiness, nausea, dizziness, headache, dyskinesia, hallucinations
Pramipexole	Treatment of signs and symptoms of Parkinson’s disease	Monotherapy in early disease; may delay development of motor complications; adjunct to levodopa in fluctuating disease; reduces OFF time; increases ON time without troublesome dyskinesia	IR: 0.125 mg daily ER: 0.375 mg daily Increase every 5–7 days to maximum 4.5 mg daily	Impulse control disorders, excessive and sudden daytime sleepiness, nausea, dizziness, headache, dyskinesia, hallucinations
Rotigotine (transdermal patch)	Treatment of signs and symptoms of Parkinson’s disease	Adjunct to levodopa in fluctuating disease; reduces OFF time; increases ON time without troublesome dyskinesia	2 mg/24 h, increase gradually to maximum 8 mg/24 h	Impulse control disorders, excessive and sudden daytime sleepiness, nausea, dyskinesia, hallucinations, application site reactions, edema, headache
Apomorphine SC injection	Acute, intermittent treatment of OFF episodes	As needed, intermittent treatment of OFF episodes, early morning OFF	2 to 6 mg, injections up to 5 times per day	Yawning, somnolence, dyskinesia, dizziness/postural hypotension, rhinorrhea, nausea, hallucinations, edema
Apomorphine sublingual film	Acute, intermittent treatment of OFF episodes	As needed, intermittent treatment of OFF episodes	10 to 35 mg, up to 5 times per day	Nausea, somnolence, dizziness, and oropharyngeal effects
MOA-B inhibitors				
Selegiline	Adjunct in the management of PD treated with levodopa/carbidopa with wearing off	Adjunct to levodopa in fluctuating disease; reduces OFF time; increases ON time without troublesome dyskinesia	Capsules 5 mg twice daily; ODT 1.25 to 2.5 mg daily	Dizziness, nausea, insomnia, headache, dyskinesia
Rasagiline	Treatment of Parkinson’s disease	Monotherapy in early disease; adjunct to levodopa in fluctuating disease; reduces OFF time; increases ON time without troublesome dyskinesia	0.5 mg daily to 1 mg daily	Dizziness, nausea, insomnia, headache, dyskinesia
MAO-B inhibitor plus channel inhibitor				
Safinamide	Adjunctive treatment to levodopa/carbidopa in patients with OFF episodes	Adjunct to levodopa in fluctuating disease; reduces OFF time; increases ON time without troublesome dyskinesia	50 to 100 mg daily	Dyskinesia, falls, nausea, insomnia
Zonisamide (off-label in the USA)	N/A	Adjunct to levodopa in fluctuating disease; reduces OFF time.	25 mg daily, increase to 50 mg or 100 mg daily	Somnolence, constipation, increased risk for renal stones
COMT inhibitors				
Entacapone	Adjunct to levodopa/carbidopa to treat wearing off	Adjunct to levodopa in fluctuating disease; reduces OFF time; increases ON time without troublesome dyskinesia	200 mg with each dose of levodopa, maximum 1600 mg per day; available as combination CLE 25 mg/100 mg/200 mg tablets	Dyskinesia, nausea, diarrhea
Opicapone	Adjunct to levodopa in patients experiencing OFF episodes	Adjunct to levodopa in fluctuating disease; reduces OFF time; increases ON time without troublesome dyskinesia	25 to 50 mg at bedtime	Dyskinesia, constipation, elevated creatine kinase, hypotension/syncope, and decreased weight
Tolcapone	Adjunct to levodopa/carbidopa for the treatment of PD^c^	Adjunct to levodopa in fluctuating disease; reduces OFF time; increases ON time without troublesome dyskinesia; liver enzyme monitoring required	100 mg three times daily; increase to 200 mg three times daily as needed	Dyskinesia, nausea, diarrhea, insomnia; rare severe hepatotoxicity
Adenosine 2A receptor antagonist				
Istradefylline	Adjunct to levodopa/carbidopa in patients experiencing OFF episodes	Adjunct to levodopa in fluctuating disease; reduces OFF time; increases ON time without troublesome dyskinesia (40 mg/day)	20 mg daily; increase to 40 mg daily as needed	Dyskinesia, dizziness, constipation, nausea, hallucinations, insomnia
NMDA receptor antagonist				
Amantadine IR	Treatment of signs and symptoms of parkinsonism	Monotherapy in early disease; adjunct to levodopa; reduces dyskinesia	Starting dose 50–100 mg daily; increase up to 100 mg three times daily	Nausea, dizziness/lightheadedness, insomnia; hallucinations, livedo reticularis, orthostatic hypotension
Amantadine ER capsules (Gocovri™)	Treatment of dyskinesia in patients with PD taking levodopa-based therapy, with or without other dopaminergic medications	Adjunct to levodopa; reduces dyskinesia; reduces OFF time; increases ON time without troublesome dyskinesia	137 mg at bedtime; can increase to 274 mg at bedtime	Hallucinations, dizziness, dry mouth, edema, constipation, falls, orthostatic hypotension
Amantadine ER tablets (Osmolex ER™)	Treatment of Parkinson’s disease	Monotherapy in early disease; adjunct to levodopa	129 mg in the morning; can increase to maximum 322 mg in the morning	Nausea, dizziness/lightheadedness, insomnia, hallucinations
Serotonin 5HT-2A and dopamine receptor antagonist				
Clozapine	N/A	Reduces dyskinesia; may reduce tremor; Monitor white blood cell count and absolute neutrophil count	12.5 to 25 mg at bedtime	Sedation, sialorrhea, rare agranulocytosis
Infusion therapies				
Carbidopa/levodopa enteral suspension	Treatment of motor fluctuations in patients with advanced PD	Reduces OFF time; increases ON time without troublesome dyskinesia; may reduce ON time *with* troublesome dyskinesia	Dose titrated per protocols; maximum daily dose of 2000 mg levodopa over 16 h per day	Tube and tube-site complications, nausea, edema
Continuous subcutaneous apomorphine infusion	N/A	Reduces OFF time, increases ON time without troublesome dyskinesia	Infusion rate of 3–8 mg per hour over 16 h per day	Infusion site and skin reactions, nausea, somnolence

^a^Adapted from 2018 International Parkinson and Movement Disorders Society Evidence-Based Medicine Review, in addition to RCT data if not included in that review

^b^Adapted from FDA prescribing information, clinical experience, and RCT data

^c^Per FDA prescribing information, tolcapone should only be used when it is clearly effective; it should be discontinued in patients who fail to show substantial clinical benefit within 3 weeks of initiation of treatment

### Carbidopa and Levodopa Extended Release Capsules (IPX066, Rytary™)

Carbidopa and levodopa extended release (CD-LD ER) is an oral formulation of levodopa designed to combine both immediate release and extended release pharmacokinetics, allowing for less frequent dosing and more stable and longer lasting plasma concentrations of levodopa compared to other formulations of oral levodopa. CD-LD ER capsules contain 4 varieties of beads: one with immediate release carbidopa-levodopa, two with different extended release carbidopa-levodopa formulations, and a fourth with an active excipient containing tartaric acid to facilitate enteral absorption [47].

Pharmacokinetic studies have demonstrated that CD-LD ER provides a rapid rise in plasma levodopa concentration with prolonged duration relative to other oral levodopa formulations [48]. In an open-label, randomized crossover study of CD-LD ER and CD-LD IR, the time to *C*_max_ was similar for both drugs (0.78 h *vs* 0.74 h, respectively), but the duration of levodopa concentration above 50% of *C*_max_ was 2.6 h longer for CD-LD ER *versus* CD-LD IR. Following a single dose, improvements in UPDRS part III scores were similar for both medications up to 2 h post-dosing, but for hours 3 through 6 UPDRS part III scores were significantly more improved with CD-LD ER than CD-LD IR. According to clinicians’ ratings, at 6 h, 68% of subjects were rated as ON without troublesome dyskinesia after taking CD-LD ER compared to 4% after taking CD-LD IR [49].

The phase 3, double-blind, randomized, controlled clinical trial ADVANCE-PD enrolled 471 patients with PD and motor fluctuations with at least 2.5 h of OFF time per day. Patients underwent a 3-week CD-LD IR dose optimization period followed by a 6-week CD-LD ER optimization period. Patients then entered a 13-week randomized, double-dummy maintenance period in which they received either their optimized CD-LD IR regimen or their optimized CD-LD ER regimen and placebo for the other. Based on home diaries, OFF time and ON time without troublesome dyskinesia both improved significantly more with CD-LD ER compared to CD-LD IR (mean treatment differences of − 1.17 h OFF time and + 0.93 h ON time without troublesome dyskinesia, *p* < 0.0001 and *p* = 0.0002 respectively). These benefits were obtained with a mean of 3.6 doses of ER per day compared to 5.0 doses of IR per day (*p* < 0.0001). The mean daily levodopa dose of ER was approximately twice that of the IR group (1630 mg *vs* 814.5 mg) [50].

Another phase 3 study (ASCEND-PD) used a randomized, double-blind, double-dummy crossover design to compare CD-LD ER to CD-LD IR plus entacapone in patients with motor fluctuations. Ninety-one patients on CD/LD IR plus entacapone were enrolled in the study and underwent open-label conversion to CD-LD ER. Patients then underwent, in randomized order, two 2-week treatment periods (separated by 1 week) with CD-LD ER or CD-LD IR plus entacapone with placebo for the other. Compared with CD-LD IR plus entacapone, patients receiving CD-LD ER experienced 1.4 h less OFF time and 1.4 h greater ON time without troublesome dyskinesia (both *p* < 0.0001) [51].

During the open-label conversion from CD-LD IR to CD-LD ER in the ADVANCE-PD trial, 5% of patients withdrew due to adverse events and 3% withdrew due to lack of efficacy. The most common adverse events during this conversion were dyskinesia (6%), nausea (5%), headache (4%), and dizziness (4%).

In patients with swallowing difficulty, CD-LD ER capsules can be opened and sprinkled onto applesauce without affecting the pharmacokinetics. Taking CD-LD ER with a high-fat, high-calorie meal delays absorption by 1 to 2 h, slightly reduces *C*_max_, and slightly increases the extent of absorption [52].

Available dosage forms for carbidopa/levodopa ER include 23.75 mg/95 mg, 36.25 mg/145 mg, 48.75 mg/195 mg, and 61.25 mg/245 mg [53]. It is important to recognize that dosing conversion from CD-LD IR to CD-LD ER is not 1:1. At the end of the 6-week open-label conversions to CD-LD ER from pre-study levodopa regimens for ADVANCE-PD and ASCEND-PD, final mean dose ratios of levodopa were largely between 2.1 and 2.4 for CD-LD IR and between 2.4 and 2.8 for CD-LD IR plus entacapone. Ratios tended to be higher for those patients taking lower daily doses of levodopa at baseline [54]. Suggested CD-LD ER dosing conversion strategies have included using approximately 3 times each individual CD-LD IR dose approximately 2/3 as often to achieve about twice the daily LD IR dose [55]. Further adjustments are likely to be required based on clinical response and early feedback, typically within 1–3 days [55]. A conservative strategy may be to convert only the morning dose to CD-LD ER, with remaining doses taken as CD-LD IR beginning when the morning dose wears off; subsequent adjustments and ultimately complete conversion are then guided by the patient’s response to the morning dose [55, 56].

### Carbidopa and Levodopa Controlled Release (Sinemet CR)

A controlled release formulation of carbidopa/levodopa (Sinemet CR, CD-LD CR) is also available. Four randomized, placebo-controlled studies of CD-LD CR *versus* CD-LD IR showed that although patients were able to reduce the number of medication administrations per day, there were no significant differences in motor response, including OFF time, between IR and CR CD-LD [57–61]. In a more recent pharmacokinetic study, the plasma levodopa concentration-time profile of CD-LD CR was only marginally shifted to the right relative to CD-LD IR, with values for *T*_max_ and duration of time during which levodopa concentrations are above 50% of *C*_max_ both about 30 min longer than those for CD-LD IR [48]. Overall, due to inconsistent absorption and pharmacokinetics, CD/LD CR does not provide significant advantages over CD/LD IR with respect to addressing motor complications, though some patients find nocturnal dosing to be helpful for nighttime symptoms. CD-LD CR is available as 25 mg/100 mg and 50 mg/200 mg tablets and is typically dosed in two or three divided doses daily [62].

### Orally Inhaled Levodopa

In 2018, the FDA approved levodopa inhalation powder (Inbrija™, CVT-301, inhaled levodopa) for the intermittent treatment of OFF episodes in patients with Parkinson’s disease treated with carbidopa/levodopa. Inbrija is a dry powder formulation of levodopa that is delivered via oral inhalation by a breath-actuated inhaler. The systemic delivery of levodopa via the pulmonary vasculature avoids many factors that contribute to a delayed or unpredictable response associated with oral ingestion of levodopa, e.g., slowed gastric transport, decarboxylation of levodopa in the gastrointestinal tract, and competition with food for jejunal absorption via active transport. Forty-two milligrams of inhaled levodopa powder provides a respirable fine-particle dose (FPD) of 25 mg (i.e., the dose estimated to reach the lungs). In a phase 2a study of patients with PD and motor fluctuations, inhaled levodopa at both 25 mg and 50 mg FPDs produced rapid rises in plasma concentration of levodopa (median time to maximum plasma concentration 15 min, compared to 66 min following oral carbidopa/levodopa) with lower between-patient variability in plasma levodopa concentrations compared to oral levodopa [63]. A subsequent 4-week, randomized, double-blind, placebo-controlled, phase 2b trial found that as assessed in clinic at week 4, inhaled levodopa 50 mg FPD administered during an OFF episode provided a UPDRS part III improvement of 7.0 points at 30 min post-dose compared to placebo (*p* < 0.001). Home diaries indicated that patients randomized to 50 mg FPD inhaled levodopa (up to 5 times per day) experienced reductions in OFF time (treatment effect − 0.9 h per day compared to placebo; *p =* 0.045) without significant increases in ON time with dyskinesia [64].

In a phase 3, 12 week randomized, double-blind, placebo-controlled trial, patients were randomized to inhaled levodopa 84 mg, 60 mg, or placebo (3). When administered during an OFF episode in-clinic at 12 weeks, both the 60 mg and 84 mg doses of inhaled levodopa produced significant reductions in UPDRS part III scores relative to placebo 30 min post-dose (mean differences of − 3.07 and − 3.92, respectively). For the 84 mg dose, there was evidence of onset of action at 10 min. Home diaries did not show significant differences in OFF time between inhaled levodopa and placebo at 12 weeks, although patients did not administer medication as often as allowed. Patients reported 3.5 OFF periods per day at baseline but only administered inhaled levodopa 84 mg approximately twice daily despite being allowed up to 5 administrations per day [65].

In these phase 2 and 3 studies, home dosing was not allowed for early morning OFF periods (before the first dose of oral carbidopa/levodopa). This was because of concern that administration without carbidopa, after not having taken carbidopa since the previous day, would increase the risk of adverse effects mediated by peripheral decarboxylation of levodopa. However, in a study randomizing patients to either a single dose of 84 mg inhaled levodopa or placebo immediately following their first morning dose of oral carbidopa/levodopa, there were no apparent increases in treatment-related adverse events including symptomatic orthostatic hypotension, nausea, or dyskinesia. An exploratory efficacy assessment found that median time-to-ON was 25.0 min following oral CD-LD IR+ inhaled levodopa *versus* 35.5 min following oral CD-LD IR+ inhaled placebo [66].

The most common side effects of inhaled levodopa include cough, nausea, upper respiratory tract infection, and discolored sputum. A study evaluating pulmonary safety found that there were no significant differences in FEV1, FVC, and FEV1/FVC ratios between those treated with inhaled levodopa *versus* inhaled placebo over 4 weeks of treatment [67]. In a 12-month prospective, randomized observational cohort study, no clinically significant changes in pulmonary function measures were seen in patients randomized to inhaled levodopa *versus* the control cohort [68]. Inhaled levodopa is not recommended for patients with chronic respiratory diseases such as asthma or chronic obstructive pulmonary disease. Each inhaled levodopa capsule contains 42 mg levodopa and the usual dosage is 2 capsules (84 mg) as needed when symptoms of an OFF period start to return, up to 5 times per day [69].

### Dopamine Agonists

Dopamine agonists (DAs) have a long history of use in PD as both monotherapy in early disease and as adjuncts to levodopa in advanced disease. Due to risks associated with earlier ergot-derived dopamine agonists, including valvular fibrosis, these agents (bromocriptine, pergolide, lisuride, and cabergoline) are no longer widely used. The non-ergot-derived DAs, including ropinirole, pramipexole, and the rotigotine transdermal patch, have all been evaluated in large, randomized, placebo-controlled, double-blinded studies and are approved for the treatment of both early and advanced PD with motor fluctuations [70].

#### Ropinirole

Ropinirole is a selective nonergoline D2/D3 receptor agonist. In one phase IIb randomized, placebo-controlled trial, 46 patients with PD and motor fluctuations requiring between 4 and 6 doses of levodopa daily were randomized to the addition of placebo or ropinirole up to 4 mg twice daily. Although percent of daily waking OFF time was reduced in the ropinirole group *versus* placebo for completers (− 50% *vs* − 20%, *p* = 0.039), this reduction did not reach statistical significance in the intention-to-treat population (− 44% *vs* − 24%, *p* = 0.085). However, the clinician’s global evaluation of change significantly favored ropinirole (78% improved in the ropinirole group compared to 35% in the placebo group, *p* = 0.004) [71]. In another randomized, placebo-controlled, double-blind trial, 149 patients with PD and predictable motor fluctuations were randomized to placebo or ropinirole dosed from 3 mg/day to a maximal dose of 24 mg/day in 3 divided doses. Levodopa dose reductions were structured with increases in ropinirole doses and were also allowed for dopaminergic adverse effects. Thirty-five percent of patients taking ropinirole experienced both at least a 20% reduction in daily OFF time and 20% reduction in levodopa dose, compared with 13% of patients taking placebo (*p* = 0.002). The reduction in levodopa dose remained significant when eliminating dose reductions due to adverse effects [72]. A ropinirole 24-h prolonged release formulation (ropinirole PR) was evaluated in a phase III randomized, placebo-controlled, double-blind study (EASE-PD). Three hundred ninety-three patients with PD and motor fluctuations with at least 3 h of OFF time per day were randomized 1:1 to the addition of placebo or ropinirole PR dosed between 2 and 24 mg/day, guided by therapeutic response and adverse effects. Patients treated with ropinirole PR experienced significant improvements in daily OFF time (mean treatment difference of − 1.7 h, *p* < 0.0001) and ON time without troublesome dyskinesia (mean treatment difference of + 1.5 h, *p* < 0.0001). Adverse events were more common with ropinirole PR, with the most frequent being dyskinesia, nausea, dizziness, somnolence, hallucinations, and orthostatic hypotension [73]. Ropinirole is typically started at 0.25 mg TID for immediate release and 2 mg daily for PR, and increased in weekly increments to a maximum daily dose of 24 mg per day. Dosing adjustment is not required for moderate renal impairment, but in end-stage renal disease the maximum recommended daily dosage is 18 mg per day [74, 75].

#### Pramipexole

Pramipexole is a nonergoline selective D2/D3 receptor agonist. In one randomized, placebo-controlled, double-blind study, 360 patients with PD and motor fluctuations were randomized to the addition of placebo or pramipexole, up to 4.5 mg per day in three divided doses. In addition to statistically significant reductions in UPDRS part II and III scores, OFF time was reduced by 31% in the pramipexole group compared to 7% in the placebo group (*p =* 0.0006) [76]. In a subsequent randomized, double-blind, placebo-controlled study, 354 patients with PD and motor fluctuations were randomized to the addition of placebo or pramipexole up to 4.5 mg per day in three divided doses. UPDRS part II and III scores were significantly improved by pramipexole (*p* = 0.0001 for both comparisons *vs* placebo), and patients in the pramipexole group experienced a reduction in daily OFF time of approximately 2.5 h compared to placebo (*p* = 0.0001) [77]. A 24-h extended release formulation of pramipexole (ER) was evaluated in a phase III randomized, placebo-controlled, double-blind trial. Five hundred seventeen patients with PD and motor fluctuations with at least 2 h of daily OFF time were randomized in 1:1:1 fashion to the addition of placebo, pramipexole ER, or pramipexole IR at doses ranging from 0.375 to 4.5 mg daily of pramipexole (daily for ER and TID for IR). Pramipexole ER reduced daily OFF time by 2.1 h compared to 1.4 h with placebo (*p* = 0.0199), and combined UPDRS part II and III scores were improved by 11.0 points by pramipexole ER compared to 6.1 points with placebo (*p =* 0.0001). ON time without troublesome dyskinesia was increased by 14.1% in the pramipexole ER group compared to 9.7% for placebo (*p* = 0.0191). Improvements with pramipexole ER were similar to those seen with pramipexole IR [78]. The most common adverse events seen in patients with advanced PD include dyskinesia, nausea, constipation, hallucinations, headache, and anorexia. Pramipexole is typically started at 0.375 mg per day (once daily for ER and in 3 divided doses for IR) and can be increased incrementally every 5 to 7 days to a maximum dose of 4.5 mg/day. Pramipexole must be dose-reduced in patients with renal impairment, and the ER formulation is not recommended for patients with severe renal impairment (creatinine clearance < 30 mL/min) or those on hemodialysis [79, 80].

#### Transdermal Rotigotine

Rotigotine is a nonergoline D3/D2 and D1 receptor agonist that is formulated for delivery via transdermal patch applied to the skin every 24 h. In one phase III randomized, placebo-controlled, double-blind study, patients with PD and at least 2.5 h of OFF time per day were randomized to the addition of placebo, rotigotine 8 mg/24 h, or rotigotine 12 mg/24 h. Mean daily OFF time was improved significantly by both rotigotine 8 mg/24 h and 12 mg/24 h compared to placebo (− 2.7 h and − 2.1 h *vs* − 0.9 h, *p* < 0.0001 and *p* = 0.0031, respectively). Similarly, ON time without troublesome dyskinesia was significantly improved (+ 3.5 h and + 2.2 h *vs* + 1.1 h, *p* < 0.0001 and *p* = 0.0078, respectively) [81]. A second trial employed a double-dummy, double-blind, randomized design to compare the addition of placebo, pramipexole, and transdermal rotigotine in PD patients with at least 2.5 h of OFF time per day. Participants were randomized in a 2:2:1 fashion to rotigotine (4 mg/24 h to 16 mg/24 h), pramipexole (0.375 to 4.5 mg/day), and placebo. Both rotigotine and pramipexole significantly improved both OFF time (− 2.5 h and − 2.8 h *vs* − 0.9 h for placebo, *p* < 0.0001) and ON time without troublesome dyskinesia (+ 2.8 h and + 2.7 h *vs* + 1.4 h for placebo, *p* = 0.0003 and *p* = 0.0007, respectively) [82]. Open-label extensions of both of these studies followed participants for up to 6 years. UPDRS part II scores remained improved for between 2 and 2.5 years relative to initial baseline, at which point they began to increase above baseline, reflecting disease progression. UPDRS part III motor scores remained improved up to 5 years follow-up, although this benefit too declined in magnitude over time. The most common side effects reported included somnolence, insomnia, dyskinesia, hallucinations, and application site reactions [83]. Rotigotine transdermal patch is typically started at 2 mg/24 h or 4 mg/24 h and can be increased by 2 mg/24 h increments to a maximum dose of 8 mg/24 h. No dosage modifications are necessary for renal impairment or for moderate hepatic impairment [84].

#### Apomorphine

Apomorphine is a dopamine agonist with affinity for both D_1_- and D_2_-like receptors (subtypes D_1_, D_2_, D_3_, D_4_, and D_5_). Because of nearly complete first-pass metabolism, it must be delivered parenterally. Apomorphine is currently approved for use in the USA and Europe as an intermittent subcutaneous injection and in Europe as a continuous subcutaneous infusion [85]. Numerous early pharmacokinetic and open-label studies demonstrated that the subcutaneous administration of apomorphine alleviates OFF symptoms in PD with an effect that is equal in magnitude to that of levodopa, but with a more rapid onset and briefer duration of action [85–90].

##### Apomorphine Subcutaneous Injection

In the USA, apomorphine is approved as a subcutaneous injection for the acute, intermittent treatment of OFF episodes associated with advanced Parkinson’s disease; this application has been studied in a number of randomized, placebo-controlled, double-blind studies. In one study, 29 participants with PD and at least 2 h of OFF time were randomized to either subcutaneous apomorphine or placebo injections in addition to their standard optimized oral medications. Participants first underwent an inpatient dose titration (ranging from 2 to 10 mg, in 2 mg increments); titration was terminated at either a dose that provided an improvement in the UPDRS part III score at least 90% of that provided by an optimally dosed levodopa challenge, or at a maximum dose of 1.0 mL (10 mg of active medication, or equivalent volume of placebo). Subsequently, participants entered a 4-week outpatient monitoring phase during which they or their caregiver were instructed to administer injections for OFF periods up to 5 times per day, but not within the hour following or the 15 min preceding a dose of oral medication. During inpatient dose titration, participants in the apomorphine group experienced improvements in the UPDRS part III score of 23.9 points (62%) compared to no change in the placebo group. During the 4 week outpatient phase, home diaries demonstrated that 95% of OFF periods had been successfully aborted by apomorphine injections, compared to 23% in the placebo group (*p* < 0.001), with an overall reduction from baseline in daily OFF time of 2 h compared to no change in the placebo group (*p* = 0.02) [91].

In a second study (APO302), 62 participants with advanced PD who had already been treated with apomorphine subcutaneous injections for OFF episodes for at least 3 months were randomized to one of four groups: 1) apomorphine at their typically effective dose, 2) apomorphine at their typically effective dose plus 0.2 mL (2 mg), 3) placebo injections at the same volume as their typically effective apomorphine dose, or 4) placebo at a volume equal to their typically effective apomorphine dose plus 0.2 mL. Assessments including the UPDRS part III score were collected at baseline in the OFF state and subsequently at various time points up to 90 min after dosing. Improvements in UPDRS part III scores were significantly greater for the pooled apomorphine groups compared to the pooled placebo groups at 10 and 20 min (− 19.9 *vs* − 5.6 and − 24.2 *vs* − 7.4 respectively, *p* < 0.0001 for both), but not at 90 min. Improvements in the typically effective and higher dose apomorphine groups were not significantly different from each other. These results suggested that treatment effects are maintained during longer term therapy, and that the doses needed to produce meaningful rescue from OFF periods are not likely to increase over time [92].

In a third study (APO303), 51 participants with PD and motor fluctuations underwent an open-label dose titration of apomorphine subcutaneous injections at 2 mg, 4 mg, 6 mg, 8 mg, and 10 mg. At the 4 mg step participants entered a randomized, double-blind, placebo-controlled crossover phase wherein they were given either apomorphine 4 mg or placebo followed by crossover to the alternate treatment. Dose escalation then continued until participants experienced intolerable side effects or reached the maximum dose of 10 mg, at which point they were transitioned to a 6-month open-label monitoring phase. Assessments including UPDRS part III scores were collected at 20, 40, and 90 min after dosing. Apomorphine 4 mg produced greater improvement in UPDRS part III scores at each time point relative to placebo. Improvements in UPDRS part III scores were dose dependent up to 6 mg, after which higher doses of apomorphine only caused more adverse effects [93].

An open-label study examined the use of apomorphine subcutaneous injections for the treatment of morning akinesia. Participants with PD who were identified via home diaries as having dose failures with their first morning levodopa doses (defined as time-to-ON greater than 60 min following levodopa dosing) underwent dose optimization titration with subcutaneous apomorphine injections to a maximum of 6 mg. During a 7-day open-label treatment phase, participants self-administered subcutaneous apomorphine injections instead of their typical first levodopa dose, and participants used a home diary to indicate whether they had achieved the ON state over successive 5-min intervals. The mean time-to-ON was reduced from 60.86 min with oral levodopa at baseline to 23.72 min at the end of the treatment phase with subcutaneous apomorphine, a treatment difference of 37.14 min (*p* < 0.0001); 95.5% of participants experienced an improvement in time-to-ON. Dose failures were less common with apomorphine than with levodopa (7% *vs* 46% of diary entries) [94].

The most common adverse effects of apomorphine subcutaneous injections include yawning, somnolence, dizziness, nausea, dyskinesia, orthostatic hypotension, and injection site effects. The antiemetic trimethobenzamide has been shown to effectively mitigate nausea resulting from apomorphine injections, but is generally not needed after 8 weeks of therapy; it is recommended to be started 3 days prior to initiation of therapy and continued for at least 2 months [95]. Importantly, coadministration with ondansetron is contraindicated due to the occurrence of profound hypotension and loss of consciousness. Patients considering the use of intermittent apomorphine injections should undergo structured dose titration and training in a controlled setting. Escalating doses of apomorphine are administered during the OFF state, beginning at 2 mg and increasing by 1 mg increments to a maximum dose of 6 mg. Evaluation of the motor response and adverse effects at each dose identifies a patient’s effective dose, which they may then inject into the abdomen or the thigh not more frequently than every 2 h [96, 97].

##### Apomorphine Sublingual Film (APL-130277)

A sublingually administered apomorphine film strip (APL-130277) has also been developed for the intermittent treatment of OFF symptoms, consisting of a thin bilayer film with one layer containing apomorphine and the other containing a pH buffering component. This formulation delivers apomorphine to the systemic circulation via the oral mucosa, avoiding first-pass metabolism associated with the enteral absorption of apomorphine [98]. In a phase II open-label dose-finding study, 19 patients with PD and at least 2 h of OFF time and 1 OFF episode per day were given escalating doses of apomorphine sublingual film, beginning at 10 mg, in the practically defined OFF state until a full ON was achieved, up to a maximum of 30 mg. 78.9% of patients achieved a full ON state (determined by UPDRS part III scores and investigator judgment) within 30 min of administration, with 40% of responders achieving a full ON state within 15 min. A full ON response was reproduced with confirmatory repeat dosing in greater than 90% of patients [99]. In a subsequent phase III, double-blind, placebo-controlled study, 109 patients with PD and at least 2 h of OFF time were randomized to placebo or apomorphine sublingual film. Sublingual apomorphine treatment significantly improved UPDRS part 3 scores at 30-min post-dosing relative to placebo (treatment difference of − 7.6 points, *p* = 0.0002) and increased the rate of patient self-rated full ON response within 30 min of dosing (35% *vs* 16%, *p* = 0.053). The most common adverse events included nausea, somnolence, dizziness, and oropharyngeal effects (including erythema, dry mouth, tongue pain). Mild to moderate oropharyngeal adverse events occurred in 31% of patients in the apomorphine group, leading to treatment discontinuation in 17% [98].

Apomorphine sublingual film received FDA approval in May 2020 for the acute, intermittent treatment of OFF episodes in patients with Parkinson’s disease. It is available in doses of 10 mg, 15 mg, 20 mg, 25 mg, and 30 mg, to be administered up to 5 times per day, at least 2 h apart. Its use is contraindicated in patients taking concurrent serotonin 5HT3 antagonist antiemetics due to the risk of hypotension and loss of consciousness seen with the use of subcutaneous apomorphine injections. Like intermittent subcutaneous apomorphine injections, it is recommended to initiate treatment in conjunction with the use of the antiemetic trimethobenzamide [100].

#### Adverse Effects

Dopamine agonists carry the risk of several adverse effects that mandate careful surveillance by treating clinicians. Excessive daytime sleepiness is common, and sudden onset sleep can also occur, both of which can pose significant safety risks in patients who are still driving. Impulse control disorders can be caused by both dopamine agonists and levodopa, but are more commonly associated with dopamine agonists; common manifestations include compulsive gambling, shopping, eating, and hypersexuality. Additional adverse effects include hallucinations, nausea, and peripheral edema. Careful identification and management of these adverse effects can maximize the safety and tolerability of this useful class of medication [101]. As noted above, the ergot-derived dopamine agonists have been associated with often irreversible, and sometimes fatal, fibrotic complications including pleural, pericardial, peritoneal, and valvular fibrosis.

### Catechol-*O*-Methyltransferase Inhibitors

Catechol-*O*-methyltransferase (COMT) is an enzyme present both peripherally and within the brain. Peripheral methylation of levodopa to 3-*O-*methyldopa (3OMD), a reaction catalyzed by COMT, reduces the central bioavailability of levodopa. Early pharmacokinetic and pharmacodynamic studies demonstrated that administration of a COMT inhibitor reduces plasma elimination of levodopa, increases levodopa serum area under concentration-time curve, and increases 6-fluorodopa at nigrostriatal terminals as assessed by positron emission tomography (PET) [102–106]. Three COMT inhibitors are now approved in the USA for the treatment of PD (tolcapone, entacapone, and opicapone).

#### Entacapone

Entacapone has been studied in a number of randomized, double-blind, placebo-controlled studies. In one study, 205 patients with PD and motor fluctuations were randomized to the addition of placebo or entacapone 200 mg with each levodopa dose. Percentage of waking time spent in the ON state was increased by 5% (approximately 1 h) in the entacapone group compared with placebo (*p* = 0.003); when stratified by baseline severity, patients with less ON time per day at enrollment experienced greater improvement with entacapone [107]. In another study, 301 patients with PD and motor fluctuations were randomized to the addition of placebo or entacapone 200 mg with each dose of levodopa. Entacapone increased ON time (treatment difference of + 0.8 h, *p* < 0.05) and reduced OFF time (treatment difference of − 0.7 h, *p* < 0.05) relative to placebo. A subgroup analysis found that patients taking 5–10 levodopa doses per day (presumably representing more severe fluctuations) experienced greater improvements in ON and OFF time (treatment differences of + 1.2 h and − 0.9 h, respectively, *p* < 0.005 for both measures) [108]. Another study randomized 171 patients with PD and motor fluctuations to placebo or entacapone 200 mg with each levodopa dose. Entacapone increased ON time and decreased OFF time relative to placebo (treatment differences of + 1.2 h and − 1.3 h respectively, *p* < 0.001 for both) [109].

A study comparing the effect of administering entacapone simultaneously with levodopa *versus* after a 30-min delay from levodopa administration found that, for advanced PD patients who had not responded well to the coadministration of the two medications, delayed administration of entacapone provided improved area under the time curve of levodopa, increased ON time, and improved UPDRS part III scores, suggesting such a delay as a possible troubleshooting strategy [110].

Entacapone is dosed 200 mg with each dose of levodopa, up to a maximum of 8 times per day (1600 mg per day). Adverse effects include dyskinesia, urine discoloration, diarrhea, nausea, hyperkinesia, abdominal pain, vomiting, and dry mouth [111].

#### Opicapone

Opicapone is a novel third generation long-acting COMT inhibitor. Once daily opicapone provided greater increases in levodopa trough levels, area under the concentration-time curve, and half-life, when compared with entacapone during coadministration with immediate release carbidopa-levodopa in healthy volunteers in a phase II randomized, parallel-group, double-blinded study [112].

The BIPARK I study was a phase III randomized, double-blinded, placebo- and active-controlled study in 600 patients with PD and motor fluctuations. Patients were randomized in a 1:1:1:1:1 fashion to placebo, entacapone 200 mg with each dose of levodopa, opicapone 5 mg daily, 25 mg daily, or 50 mg daily. Opicapone 50 mg reduced daily OFF time from baseline by 60.8 min relative to placebo (*p =* 0.0015, fulfilling superiority to placebo) and by 26.2 min relative entacapone (*p* = 0.0051, fulfilling noninferiority to entacapone). In addition, opicapone 50 mg daily increased total ON time (+ 71.9 min *vs* placebo, *p* = 0.0001) as well as ON time without troublesome dyskinesia (+ 62.26 min *vs* placebo, *p* = 0.002), without a significant increase in ON time with troublesome dyskinesia [113].

The BIPARK II study randomized 427 patients with PD and motor fluctuations to placebo, opicapone 25 mg daily, or opicapone 50 mg daily. Opicapone 50 mg daily reduced daily OFF time by 54.3 min relative to placebo (*p* = 0.008) and increased daily total ON time by 52.6 min relative to placebo (*p* = 0.005). ON time with troublesome dyskinesia was not significantly different in either opicapone dosage group compared with placebo. Two hundred eighty-six of the patients completed a 1-year open-label extension, during which these benefits were maintained [114]. In both studies, dyskinesia was the most frequent treatment-related adverse event, occurring in 16–24% of patients taking 50 mg opicapone.

A pooled safety analysis of BIPARK I and BIPARK II showed that the most common treatment-emergent adverse events for opicapone 50 mg, opicapone 25 mg, and placebo were dyskinesia (20.4%, 16%, and 6.2%, respectively), constipation (6.4%, 4.9%, and 1.9%, respectively), and insomnia (3.4%, 7%, and 1.6%, respectively). No significant changes in laboratory values (including liver function tests), ECG parameters, or vital signs were noted through the double-blind or 1-year open-label extension periods [115, 116].

Opicapone was approved by the FDA in April 2020 as an adjunctive treatment to levodopa/carbidopa in patients with Parkinson’s disease experiencing OFF episodes. It is available in doses of 25 mg and 50 mg capsules, with a recommended dose of 50 mg at bedtime, or 25 mg at bedtime in patients with moderate hepatic impairment [117].

#### Tolcapone

Tolcapone is a selective and reversible COMT inhibitor with activity in both the periphery and in the brain. A number of randomized, double-blind, placebo-controlled studies have demonstrated that the use of tolcapone in conjunction with levodopa plus a decarboxylase inhibitor (carbidopa or benserazide) leads to reductions in daily OFF time, increases in daily ON time, and decreases in total daily levodopa dosage. In one study, 202 patients with PD and motor fluctuations taking levodopa were randomized to placebo, tolcapone 100 mg three times daily (TID), or tolcapone 200 mg TID. After 12 weeks of therapy, OFF time was reduced by 3.2 h per day in the tolcapone 200 mg TID group *versus* a reduction of 1.4 h per day in the placebo group (*p* < 0.01); the reduction of 2.3 h per day in the tolcapone 100 mg TID group was not statistically significant. Reductions in the daily levodopa dose in both treatment groups were significant relative to placebo [118]. In another study, 177 patients with PD and motor fluctuations were randomized to placebo, tolcapone 100 mg TID, or tolcapone 200 mg TID [7]. Reduction in OFF time at 12 weeks was greater in both tolcapone groups than in the placebo group, but this difference was only statistically significant for the 100 mg TID group (− 12.7% of day *vs* − 4.2% of day, *p* < 0.05). Increases in ON time were significant for both tolcapone dosage groups (+ 10.8% of the day in both tolcapone groups *vs* − 0.07% for placebo, *p* < 0.01), and total daily levodopa dose was decreased in both tolcapone groups relative to placebo [119]. A third study randomized 215 patients with PD and motor fluctuations to placebo or tolcapone 100 mg TID or 200 mg TID and found significant reductions in OFF time, increases in ON time, and reduced total levodopa daily doses for both tolcapone dosages [120].

Concerns regarding potentially fatal hepatotoxicity associated with tolcapone (and consequent strict monitoring requirements) have limited its widespread use. In the above phase III studies of tolcapone, elevations of serum alanine aminotransferase (ALT) and aspartate aminotransferase (AST) above 3 times the upper limit of normal were seen in approximately 1–3% of patients, although no cases of hepatic failure were observed. Following initial approval, 4 cases of severe hepatic dysfunction, with 3 of these resulting in death, were reported, prompting suspension of marketing of tolcapone in Europe and Canada and the institution of more stringent prescribing and monitoring guidelines in the USA [121]. However, all cases occurred within 6 months of initiating therapy with tolcapone, and the recommended schedule of liver function testing was not followed in any of these cases. Subsequent postmarketing surveillance found only 3 cases of severe liver enzyme elevation which were all reversible, and no deaths, prompting reintroduction of the drug in Europe and relative relaxation of monitoring guidelines in the USA [122]. A subsequent observational study conducted between 2005 and 2009 of 391 patients treated with tolcapone found that 8.7% of patients experienced an elevation of AST, ALT, or both; only 5 patients had elevations greater than twice the upper limit of normal, LFT elevations were reversible in all cases, and a majority of cases occurred within the first 6 months of treatment [123]. In the USA, monitoring of AST and ALT is recommended every 2 to 4 weeks for the first 6 months of treatment, followed by periodic monitoring at intervals deemed clinically relevant. Tolcapone should be stopped if AST or ALT rise above twice the upper limit of normal, or if clinical signs of liver disease develop.

The most common adverse effects with tolcapone include dopaminergic effects such as dyskinesia, nausea, and insomnia, with diarrhea the most common non-dopaminergic effect. The starting dose is 100 mg three times daily, which can be increased to 200 mg three times daily if needed. The first dose should be taken with the first dose of levodopa, with subsequent doses taken at intervals of 6 to 8 h. The dose of levodopa may need to be decreased upon the introduction of tolcapone or shortly thereafter in order to reduce dyskinesia [124]. The rare risk of fatal hepatotoxicity has greatly limited its use in clinical practice.

#### Comparative Studies

The direct comparative efficacies of entacapone and tolcapone were assessed in a double-blind, randomized, active-controlled switch trial in which patients with PD and motor fluctuations taking entacapone were randomized to either continue their current regimen or replace entacapone with tolcapone 100 mg TID. The percentage of patients experiencing an increase in ON time of ≥ 1 h per day was numerically greater in the tolcapone group than in the entacapone group (58% *vs* 47%), but this difference was not statistically significant (*p =* 0.21). The results of selected exploratory outcomes, however, favored tolcapone: patients taking tolcapone experienced greater mean increases in ON time (1.34 h *vs* 0.65 h), and a greater proportion of patients taking tolcapone experienced increases in ON time > 3 h compared to those treated with entacapone (29% *vs* 12%) [125]. In another study, 40 patients with PD and motor fluctuations who had been treated with tolcapone before it was discontinued either due to side effects or to regulatory indications by the European drug authority were prospectively started on entacapone for 3 months followed by withdrawal of entacapone. Increases in ON time and decreases in OFF time were greater during treatment with tolcapone compared to entacapone (+ 15% *vs* + 8% and + 16% *vs* + 7%, respectively, with *p* = 0.01 for tolcapone *vs* baseline and *p* = 0.05 for tolcapone *vs* entacapone) [126]. A systematic review and meta-analysis examined 14 randomized, placebo-controlled trials of tolcapone or entacapone and found that although both tolcapone and entacapone were statistically superior to placebo, the weighted mean differences for improvements in placebo-corrected ON time and OFF time in the tolcapone-treated patients were greater than those in the entacapone-treated patients (+ 1.86 h *vs* + 1.02 h and − 1.60 h *vs* − 0.68 h, respectively) [127]. In an open-label extension study of the BIPARK I trial, patients who switched from entacapone to opicapone (at the beginning of the open-label phase) experienced an improvement in OFF time of 39.3 min compared to their open-label baseline (*p* = 0.0060) [128].

#### Carbidopa/Levodopa/Entacapone (Stalevo)

Carbidopa/levodopa/entacapone (CLE) is a combination formulation of carbidopa, levodopa, and entacapone. FDA approval was granted based upon studies evaluating entacapone as add-on to carbidopa-levodopa [107, 109]. In one phase III open, parallel-group, active treatment-controlled study, 176 patients with PD and end-of-dose wearing OFF were randomized to either CLE or CD-LD IR plus entacapone. Clinical outcomes were not significantly different between the two groups, but 81% of patients preferred CLE compared to two separate tablets [129]. In a study of 62 patients with PD and motor fluctuations taking CD-LD CR who were switched to CLE, 42 patients preferred CLE whereas 20 patients preferred CD-LD CR [130]. In another study, 52 patients switched to CLE from immediate-release carbidopa-levodopa; 86% of patients were able to switch their entire regimen to CLE, and patients found CLE to be simpler and more convenient to dose, easier to remember, and easier to swallow than their prior medication regimen [131]. CLE is available in a range of dose combinations, with carbidopa:levodopa in 1:4 ratio, combined with 200 mg entacapone. Adverse effects include dyskinesia, urine discoloration, diarrhea, nausea, abdominal pain, vomiting, and dry mouth [132].

### Selective Monoamine Oxidase Type B Inhibitors

Monoamine oxidase inhibitors reduce the enzymatic degradation of dopamine within the synapse, thereby increasing its concentration and extending the time over which it can activate postsynaptic dopamine receptors. Monoamine oxidase exists in two isoforms: monoamine oxidase type A (MOA-A) and monoamine oxidase type B (MAO-B). MOA-A is present both centrally and peripherally. Peripherally, MAO-A deactivates circulating catecholamines as well as the dietary vasopressor tyramine. Therefore, both MAO-A selective and non-selective MAO inhibitors pose a risk of hypertensive crisis when combined with dietary ingestion of tyramine rich foods (e.g., hard cheeses). Selective inhibition of MAO-B avoids these effects [133, 134]. To date, three selective MAO-B inhibitors are approved for the treatment of Parkinson’s disease: selegiline, rasagiline, and safinamide.

#### Selegiline

Selegiline, the first selective and irreversible MAO-B inhibitor to be approved, is available as an immediate release tablet and as an orally dissolvable tablet (ODT) as an adjunctive therapy to levodopa. Because selegiline is an irreversible inhibitor of the MAO-B enzyme, the duration of clinical effect exceeds its elimination half-life (of about 1.5 h), and decline in clinical effect after discontinuation of selegiline depends upon recovery of brain MAO-B via protein synthesis. The rate of MAO-B recovery is dose-, organ- and species-dependent; recovery of MAO-B activity occurs after 2 weeks in human platelets, whereas the half-life of recovery of MAO-B in pig brains is about 6.5 days [135–137]. Following a number of small studies suggesting that oral selegiline added to carbidopa-levodopa improved motor fluctuations, a multicenter, randomized, placebo-controlled trial of oral selegiline plus optimized carbidopa-levodopa in 99 patients found that subject diary measures of walking and overall motor disability favored selegiline over placebo (*p* = 0.002 and *p* < 0.001, respectively) [138, 139]. The ODT formulation reduces first-pass metabolism. In one randomized, double-blind, placebo-controlled study, 140 subjects with at least 3 h of OFF time per day were randomized in a 2:1 fashion to ODT selegiline at two successively increasing doses or placebo. At the end of 12 weeks, subjects in the selegiline ODT group experienced significant improvements in both OFF time and ON time without dyskinesia relative to placebo (− 2.2 h *vs* − 0.6 h, *p* < 0.001; + 1.8 h *vs* + 0.4 h, *p* = 0.006), whereas ON time with dyskinesia was not significantly different between groups [140]. However, a second identically designed trial randomizing 99 patients in a 2:1 fashion to the same escalating selegiline ODT doses or placebo found no significant differences in home diary measures of OFF time, although selegiline ODT significantly improved ON time without dyskinesia at 12 weeks (+ 1.9 h *vs* + 0.9 h, *p* = 0.035). These differences appeared to be largely due to significantly greater placebo responses in the latter trial. Selegiline ODT was approved by the FDA based on a pooled analysis of both trials finding significant improvements in the primary endpoint of percent reduction in OFF time, and an open-label extension study of selegiline ODT 2.5 mg/day showed similar sustained reductions in OFF time from baseline [141, 142]. The most common adverse effects in these studies included dizziness, nausea, insomnia, headache, and dyskinesia.

Selegiline capsules are dosed at 5 mg twice daily. Selegiline ODT is dosed initially at 1.25 mg daily and can be increased to a maximum dosage of 2.5 mg daily as determined by clinical response [143, 144].

#### Rasagiline

Rasagiline, a second-generation selective and irreversible MAO-B inhibitor, is approved by the FDA as both monotherapy and as adjunctive therapy to carbidopa/levodopa in PD. Two phase III randomized, placebo-controlled, double-blind trials, LARGO and PRESTO, established the efficacy of rasagiline in treating motor fluctuations in advanced PD.

In the LARGO study, 687 patients with PD treated with levodopa/decarboxylase inhibitor and experiencing at least 1 h of daily OFF time were randomized to the addition of rasagiline 1 mg daily, entacapone 200 mg with each levodopa dose, or placebo, for a treatment period of 18 weeks. Adjusted mean changes in OFF time from baseline (averaged over 12 home diaries for the preceding 4 study visits) were significantly improved for both rasagiline and entacapone relative to placebo (treatment differences of − 1.18 h for rasagiline and − 1.20 h for entacapone, *p* = 0.0001 and *p* < 0.0001, respectively). Additionally, rasagiline and entacapone both significantly increased daily ON time without troublesome dyskinesia (treatment differences of + 0.82 h for both, *p* = 0.0005) without significant increases in daily ON time with troublesome dyskinesia. Interestingly, rasagiline also improved the UPDRS part III scores during the practically defined OFF state (− 5.64 points relative to placebo, *p* = 0.0130), potentially owing to its irreversible MAO inhibition. Rates of adverse events were similar in each group, with no increase in treatment-related dopaminergic side effects relative to placebo observed [145].

In the PRESTO study, 472 patients with PD treated with levodopa/decarboxylase inhibitor and at least 2.5 h of daily OFF time were randomized to the addition of 0.5 mg daily of rasagiline, 1 mg daily of rasagiline, or placebo, for a treatment period of 26 weeks. Mean reductions in daily OFF time averaged over 9 home diary entries preceding 3 study visits were significantly greater for both rasagiline 0.5 mg daily and 1 mg daily (treatment differences of − 0.49 h and − 0.94 h, *p* = 0.02 and *p* < 0.001, respectively). Both dosages provided increases in ON time without dyskinesia (+ 0.51 h and + 0.78 h relative to placebo, *p* = 0.05 and *p* = 0.004, respectively), although 32% of the increase in total ON time for the 1 mg daily dose included troublesome dyskinesia. Adverse events that were more common in patients treated with rasagiline included weight loss, vomiting, anorexia, and balance difficulty [146].

As an adjunct to levodopa, rasagiline is started at 0.5 mg daily and can be increased to 1 mg daily as determined by clinical response [147].

#### Safinamide

Safinamide, an ɑ-aminoamide with both selective MAO-B inhibition and inhibition of glutamate release via voltage-dependent sodium and calcium channel antagonism, was approved by the FDA for use as an adjunctive treatment to carbidopa/levodopa in 2017. Unlike selegiline and rasagiline, it is a reversible inhibitor of MAO-B, but given its terminal half-life of approximately 22 h, it is able to be dosed once daily [148].

A phase III double-blind, placebo-controlled study randomized 669 patients with PD and at least 1.5 h of OFF time per day to the addition of safinamide 50 mg/day, safinamide 100 mg/day, or placebo. Over the 24-week study period, both safinamide 50 mg/day and 100 mg/day showed significant improvements in ON time with no or non-troublesome dyskinesia (least squares mean differences of + 0.51 h and + 0.55 h *vs* placebo, respectively; *p* < 0.05 for both), as well as significant improvements in OFF time (least squares mean differences of − 0.6 h *vs* placebo for both dosages, *p* < 0.05 for both groups). No significant changes in ON time with troublesome dyskinesia were observed [149]. In an 18-month randomized, double-blind, placebo-controlled extension study of this trial, 544 patients continued on their previous treatment. The primary efficacy endpoint was mean change from baseline to endpoint of the total score on the Dyskinesia Rating Scale (DRS) during ON time. Although reductions in the total DRS scores were observed for both groups, differences *versus* placebo were not statistically significant. However, ad hoc analysis of patients who at enrollment had moderate to severe dyskinesia (comprising 36% of the study population) found that DRS total score reductions were significantly greater in the safinamide 100 mg/day group *versus* placebo (LS mean difference *vs* placebo of − 1.5 points, *p* = 0.0317). This difference was maintained even when excluding patients who experienced a reduction in levodopa dose. Additionally, other secondary endpoints showed significant improvements in both the safinamide 50 mg/day and 100 mg/day groups, including reductions in OFF time (LS differences *vs* placebo of − 0.62 h/day and − 0.75 h/day, *p* = 0.0011 and *p* < 0.0001) and improvements in ON time without troublesome dyskinesia (LS differences *vs* placebo of + 0.67 h/day and + 0.83 h/day, *p* = 0.0031 and *p* = 0.0002 respectively) [150]. A subsequent phase 3 double-blind, placebo-controlled study randomized 549 patients with PD and at least 1.5 h of OFF time per day to safinamide 100 mg/day or placebo. ON time without troublesome dyskinesia was significantly increased in patients taking safinamide (LS mean difference *vs* placebo of + 0.96 h, *p* < 0.001), accompanied by significant reductions in OFF time (LS mean difference of − 1.03 h, *p* < 0.001). DRS scores were not significantly different in the safinamide group *versus* placebo, although UPDRS part IV scores were nominally increased (LS mean difference of + 0.26 points, *p* = 0.04) [151].

In the above trials, safinamide was generally well-tolerated; the most common adverse events included dyskinesia, falls, nausea, and insomnia. Safinamide is started at 50 mg/day and increased to 100 mg/day after 2 weeks based on individual need and tolerability. It should be dose-reduced in patients with moderate hepatic impairment and is contraindicated in those with severe hepatic impairment. No adjustment is needed for impaired renal function [152].

#### Zonisamide

Zonisamide, an antiseizure medication, has been shown to have beneficial effects on the motor symptoms of PD [153–156]. Zonisamide exhibits inhibition of MAO-B, as well as inhibition of sodium and calcium channels, reduces D1-receptor associated GABA transmission, and activates dopamine synthesis and release [153].

A phase 3 double-blind, placebo-controlled trial randomized 389 patients with PD and at least 2 h of daily OFF time to the addition of placebo, zonisamide 25 mg daily, or zonisamide 50 mg daily. Zonisamide 50 mg daily significantly reduced daily OFF time compared to placebo (mean treatment difference of − 0.709 h, *p* = 0.005), without significant increases in dyskinesia. Adverse effects occurring more frequently with zonisamide than placebo were somnolence and constipation [153]. From clinical experience and use in the treatment of epilepsy, zonisamide is known to increase risk for renal stones. Dosing in the above trials ranged from 25 mg daily to 100 mg daily, though doses as high as 600 mg daily are used for epilepsy [157]. Although zonisamide holds regulatory approval in Japan for the treatment of PD, its use in the USA for PD is currently off-label.

### Istradefylline

Istradefylline is an adenosine A2A receptor antagonist approved in Japan in 2013 and approved in the USA in 2019 as adjunctive treatment to levodopa/carbidopa in patients with PD experiencing OFF episodes. In the face of dopamine deficiency, adenosine A2A antagonists exert antiparkinsonian activity by attenuating overactivity of striatopallidal neurons and by decreasing excessive GABAergic inhibition of the globus pallidus externus, thereby returning the indirect motor pathway toward a more normal state [158, 159]. Early trials in MPTP lesioned non-human primates demonstrated that treatment with istradefylline reduced parkinsonian motor signs without causing or exacerbating levodopa-induced dyskinesia [160–162]. Approval in the USA by the FDA was based on efficacy demonstrated in four 12-week, multicenter, randomized, double-blind, placebo-controlled phase 3 trials in PD patients with OFF episodes taking stable regimens of levodopa/carbidopa and other anti-PD medications [163–166]. In these trials, istradefylline 20 mg once daily provided reductions in OFF time compared to placebo of 0.7 h (*p* = 0.03)[164], 0.65 h (*p* = 0.013)[165], and 0.76 h (*p* = 0.003)[166] and istradefylline 40 mg once daily provided reductions in OFF time compared to placebo of 1.15 h (*p* = 0.006) [163], 0.92 h (*p* < 0.001) [165], and 0.74 h (*p* = 0.003) [166]. The incidence of patients discontinuing for any adverse reaction was 5% for placebo, 5% for istradefylline 20 mg, and 6% for istradefylline 40 mg. New or increased dyskinesia was the most commonly reported adverse event, occurring in 8% of placebo patients, 15% of istradefylline 20 mg patients, and 17% of istradefylline 40 mg patients. However, only 1% of istradefylline patients discontinued due to dyskinesia. Additional adverse events included (placebo/20 mg/40 mg) dizziness (4%/3%/6%), constipation (3%/5%/6%), nausea (5%/4%/6%), hallucination (3%/2%/6%), and insomnia (4%/1%/6%).

Results from a postmarketing surveillance study in Japan indicated that the most frequent adverse drug events were dyskinesia, hallucinations, and somnolence. Clinicians rated effectiveness for reduced OFF time at 38.2%, for improved motor dysfunction at 48.5%, and overall at 61.3% [167].

The half-life of istradefylline at steady-state is approximately 83 h and it is exclusively eliminated via hepatic metabolism, primarily via CYP1A1 and CYP3A4. The recommended dosage is 20 mg once daily which can be increased to a usual maximum of 40 mg once daily. It can be taken with or without food. The maximum recommended dosage in patients with moderate hepatic impairment (Child-Pugh B) is 20 mg once daily and it should be avoided in patients with severe hepatic impairment (Child-Pugh C). In patients who smoke 20 or more cigarettes per day (or the equivalent of another tobacco product), the recommended dosage is 40 mg once daily [168].

### Amantadine (Immediate-Release, Extended Release Capsules, Extended Release Tablets)

Amantadine is a non-selective, noncompetitive glutamatergic NMDA receptor antagonist. Preclinical and animal studies have supported the hypothesis that excessive cortico-striatal glutamatergic activity can exacerbate parkinsonism during dopamine troughs and drive dyskinesia during times of dopamine peaks [169, 170]. An early double-blind, placebo-controlled, crossover study involving 18 patients found that dyskinesia scores during a steady-state optimal dose levodopa infusion were 60% lower with amantadine immediate release (IR, mean dose = 350 mg/day) compared to placebo [171]. During outpatient administration of oral levodopa, these patients also experienced significantly decreased duration and severity of dyskinesia (UPDRS IV items 32 and 33) and significantly less OFF time (UPDRS IV item 39). A 1-year follow-up study of this same patient cohort found persistent benefit of amantadine IR for reducing both dyskinesia and OFF time [172].

A number of additional small, double-blind, placebo-controlled crossover trials demonstrated reduction in dyskinesia with amantadine IR compared to placebo [173–175]. Although there have been concerns that amantadine’s antidyskinetic effect wanes over time, a number of studies contradicted this. The AMANDYSK trial randomized patients taking stable doses of amantadine IR for peak-dose LID for at least 6 months to either continued amantadine IR or placebo; over the 3-month study period, there were significant increases in dyskinesia scores in patients who discontinued amantadine, and 62% of patients in the placebo arm dropped out early due to worsening of dyskinesia [176]. A second study with a similar design randomized patients on amantadine IR for LID for at least 1 year to either amantadine or placebo and similarly found that dyskinesia scores worsened in the placebo arm during the 3-week study period [177].

Two extended release formulations of amantadine are now available. Amantadine extended release (ER) capsules (Gocovri™, ADS-5102) is approved for the treatment of levodopa-induced dyskinesia. Amantadine ER is taken at bedtime and isformulated to provide a slow rise in amantadine concentrations overnight and high concentrations in the morning and through the waking day. Amantadine ER was studied in two multicenter phase III randomized, double-blind, placebo-controlled studies: EASE LID and EASE LID 3 [178, 179]. Gocovri 274 mg daily at bedtime significantly reduced dyskinesia compared to placebo as assessed using the Unified Dyskinesia Rating Scale (UDysRS). In addition, Amantadine ER significantly decreased OFF time (− 0.9, − 1.1 h) and increased ON time without troublesome dyskinesia (+ 2.8, + 1.9 h) compared to placebo in EASE LID and EASE LID 3, respectively [178, 179]. A pooled analysis described a relative treatment difference between amantadine ER and placebo of 27.3% on UDysRS scores (*p* < 0.0001) and a reduction in daily OFF time of − 1.00 h (*p* = 0.0006) [180]. An analysis of EASE LID and EASE LID 3 home diary data that determined the mean daily number and durations of episodes of time spent in each motor state (“Good ON” [ON without dyskinesia plus ON with non-troublesome dyskinesia], ON with troublesome dyskinesia, and OFF) found that patients treated with amantadine ER experienced greater improvements in the number and duration of episodes of OFF, ON without troublesome dyskinesia, and ON with troublesome dyskinesia. In addition, patients experienced fewer transitions between states (treatment difference of − 2.2 transitions per day, *p* = 0.004). These changes were associated with improvements in cumulative OFF time (least squares mean treatment difference *vs* placebo of − 1.2 h, *p*  <  0.001), ON time with troublesome dyskinesia (least squares mean treatment difference of − 1.5 h, *p*  <  0.001), and “Good ON” time (least squares mean treatment difference of +2.5 h, *p* <  0.001) [181].

Another formulation, amantadine ER tablets (Osmolex ER™), is approved for the treatment of Parkinson’s disease and drug-induced extrapyramidal reactions in adult patients. FDA approval was based on bioavailability studies comparing amantadine ER tablets to amantadine IR. To our knowledge, no clinical trials have specifically assessed the efficacy of amantadine ER tablets in the treatment of LID.

The most common side effects of amantadine include hallucinations, dizziness, dry mouth, peripheral edema, constipation, falls, and orthostatic hypotension [180].

Amantadine IR is often started at 50 mg or 100 mg daily and increased to a typical maintenance dose of 100 mg twice daily; total daily doses of 300 to 400 mg per day may be tried, but these doses are often limited by side effects. The initial daily dosage of amantadine ER capsules is 137 mg, administered orally once daily at bedtime. This can be increased after 1 week to the recommended dosage of 274 mg once daily at bedtime [182]. The recommended initial dosage of amantadine ER tablets is 129 mg administered orally once daily in the morning, which can be increased in weekly intervals to a maximum daily dose of 322 mg (administered as a 129 mg and 193 mg tablet), taken in the morning [183]. Amantadine is primarily renally cleared, requiring adjusted dosing in patients with impaired renal function, and is contraindicated in patients with end-stage renal disease [184].

### Clozapine

Clozapine is an atypical neuroleptic with strong antagonist activity at serotonin 2A (5-HT2A) and dopamine D4 receptors but weak antagonistic activity at dopamine D2 receptors. This pharmacologic profile is responsible for clozapine’s antipsychotic benefit with low propensity to cause or worsen parkinsonism, as commonly occurs with many other dopamine receptor blocking neuroleptics. Observations from the treatment of psychosis in PD patients suggested that clozapine might hold potential to improve some motor features. An early open-label study of 6 patients with PD with motor fluctuations found that clozapine improved motor symptoms during both morning akinesia as well as interdose OFF periods, including a notable reduction in tremor severity [185]. Several other open-label studies found that treatment with clozapine, often at doses less than 200 mg per day, significantly reduced the prevalence and severity of levodopa-induced (and in one study apomorphine-induced) dyskinesia [186–189]. A randomized, double-blind, placebo-controlled trial involving 50 patients with PD and disabling LID found that clozapine, at mean doses of approximately 40 mg daily, reduced the daily duration of LID by approximately 2 h relative to placebo (*p* = 0.003), without otherwise modifying the motor response to levodopa [190]. Common side effects in the above studies included sedation and sialorrhea. The risk for severe agranulocytosis with clozapine mandates weekly laboratory monitoring for 6 months, bi-weekly for an additional 6 months, and monthly thereafter. This safety concern makes it used only rarely for LID in PD.

### Medication Device–Assisted Therapies

#### Continuous Subcutaneous Apomorphine Infusion

In addition to intermittent subcutaneous injections of apomorphine for the treatment of OFF episodes, the continuous subcutaneous apomorphine infusion (CSAI) has emerged as a useful tool in advanced PD, with substantial clinical experience in Europe in which it is approved for the management of advanced PD with motor complications. Several open-label studies have demonstrated reductions in OFF time, increases in ON time, decreases in dyskinesia, and, in many cases, reductions in daily total levodopa equivalent doses; several longitudinal and retrospective studies of long-term treatment demonstrate continued efficacy over time, albeit with appreciable rates of eventual discontinuation due to adverse effects [89, 90, 191–200]. A multicenter observational open-label study comparing outcomes following carbidopa-levodopa enteral suspension (CLES) and CSAI in participants with PD and motor fluctuations found that, at 6 months, scores on the UPDRS parts III and IV and the PDQ-8 were significantly improved from baseline in both groups, with relative changes between modalities not significantly different [197]. A subsequent observational study (EuroInf 2) comparing outcomes following CLES, CSAI, and bilateral subthalamic nucleus deep brain stimulation (STN-DBS) found similar improvements in motor complications between these groups. In addition, several nonmotor symptoms improved following each intervention; specifically mood/cognition, perceptual problems/hallucinations, and attention/memory scores in the Nonmotor Symptoms Scale for Parkinson’s disease (NMSS) improved in the CSAI group [201]. Other studies have also found a positive profile for apomorphine with respect to visual hallucinations. It has been proposed that the piperidine moiety of apomorphine, which is shared with pimavanserin and many neuroleptics and confers antagonism at 5HT2A serotonin receptors, may be partly responsible [202].

CSAI was also evaluated in a pivotal phase III, multicenter, randomized, double-blind, placebo-controlled study (TOLEDO). One hundred-seven participants with PD and motor fluctuations and at least 3 h of OFF time daily were randomized to the addition of either apomorphine or placebo subcutaneous infusion. Participants were admitted to the hospital and underwent titration of infusion rates to identify each participant’s optimal dosing rate (range of 3–8 mg/h), administered over 16 h per day. Oral medications were adjusted during this phase, allowing for the hierarchical reduction or discontinuation of dopamine agonists, MAO-B inhibitors, and finally levodopa or combined levodopa/COMT inhibitors. Dosing could be adjusted for up to 4 weeks, followed by an 8-week maintenance study phase, with participants offered transition to a final 52-week open-label phase. Participants in the apomorphine group experienced greater reductions in OFF time (− 2.47 h per day *vs* − 0.58 h per day, *p* = 0.025), greater increases in ON time without troublesome dyskinesia (+ 2.77 h per day *vs* + 0.8 h per day, *p* = 0.0008), and greater reduction in levodopa equivalent daily doses of oral medication (− 492.1 mg *vs* − 163.7 mg, *p* = 0.0014). During the treatment phase, six participants in the apomorphine group withdrew due to adverse effects, whereas none did in the placebo group. Overall, AEs were more common with apomorphine compared to placebo (92.6% *vs* 56.6%), and the most common treatment-emergent adverse effects were skin nodule formation and/or erythema at the infusion site, nausea, and somnolence [44]. CSAI is not currently FDA approved in the USA, although clinical trials are ongoing.

#### Carbidopa-Levodopa Intestinal Suspension (Duodopa/Duopa™)

Carbidopa-levodopa enteral suspension (CLES) is administered via a portable infusion pump into the jejunum through a percutaneous endoscopic gastrostomy using a jejunal tube (PEG-J) to provide continuous intestinal delivery of levodopa. CLES has been well-studied, with regulatory approval in Europe in 2004–2005 (Duodopa) and the USA in 2015 (Duopa) for the indication of treatment of motor fluctuations in patients with advanced Parkinson’s disease.

Early studies of continuous intravenous and intraduodenal infusions of levodopa demonstrated reduced fluctuations in plasma concentration in levodopa as well as improved motor performance [203–208]. A randomized crossover trial in which 12 patents with PD and motor fluctuations were randomized to optimized doses and rates of either nasaduodenal CLES or oral carbidopa/levodopa controlled release found significantly lower variations and maximums in mean plasma concentrations with CLES *versus* oral medication (14% *vs* 34%, *p* < 0.01, and 3.5 ± 1.1 μg/mL *vs* 4.6 ± 1.3 μg/mL, *p* < 0.01, respectively) [209]. Multiple open-label studies demonstrated that CLES can effect reductions in OFF time without worsening of dyskinesia relative to patients’ baseline control with oral medications [210–215].

Larger, phase 3 prospective trials found similar significant improvements in control of motor fluctuations with CLES. In a pivotal phase 3, double-blind, double-dummy, randomized study of CLES, 71 patients with advanced PD and motor fluctuations underwent placement of gastrojejunostomy tubes and were subsequently randomized to either a) over-encapsulated CD-LD IR plus placebo CLES infusion or b) CLES infusion plus over-encapsulated placebo CD-LD IR [42]. Dose titration of the rate of infusion and oral administration dosages occurred over 4 weeks, followed by an 8-week maintenance period, with open-label oral CD-LD IR available as rescue therapy for persistent OFF periods in both groups. Relative to oral levodopa, patients receiving CLES experienced greater reductions in OFF time (treatment difference of − 1.91 h/day, *p* = 0.0015), greater increases in ON time without troublesome dyskinesia (treatment difference of + 1.86 h/day, *p* = 0.0142), and greater increases in ON time without any dyskinesia (treatment difference of + 2.28 h/day, *p* < 0.05). Device-related complications occurred in 89% of patients and included intestinal tube dislocations in 24% and occlusions in 13%. Additional adverse events included tube and stoma insertion complications, pump malfunctions, and pneumoperitoneum [42]. A 52-week long open-label extension of this study found sustained clinical benefit in those who continued CLES, as well as a comparable improvement of motor fluctuations in those who were initiated on CLES at the beginning of the open-label period [216]. In these studies, improvements (reductions) in ON time with troublesome dyskinesia were not statistically significant. To more specifically examine the effects of CLES on troublesome dyskinesia, a post hoc exploratory analysis was performed in the subset of patients with more severe dyskinesia (at least 1 h of troublesome dyskinesia per day at double-blind baseline). Compared to baseline, patients in the CLES group experienced significant improvements in ON time with troublesome dyskinesia (− 1.8 h, *p* = 0.14), though the difference compared to CD-LD IR was not statistically significant. ON time without troublesome dyskinesia and OFF time were similarly improved relative to baseline (+ 4.4 h, *p* = 0.004; − 2.7 h, *p* = 0.15, respectively). Similar improvements were found when examining patients fitting these criteria in the open-label extension phase [217]. A second, large, open-label, 12-month prospective study of CLES in 354 patients found significant decreases in OFF time as well as increases in ON time without troublesome dyskinesia compared to baseline (OFF: − 4.4 h per day, *p* < 0.001; ON without troublesome dyskinesia: + 4.8 h per day, *p* < 0.001) [218].

Practices for candidate selection, initial titration and dose adjustments, and maintenance are described in a recent review article by Amjad et al. [219]. The maximum recommended daily dose of CLES is 2000 mg of levodopa (i.e., one cassette per day) administered over 16 h [220].

### Medication Therapies in Late Stage Development

A number of additional medication therapies to ameliorate motor complications in PD are under investigation; currently, the furthest advanced (under study in phase 3 clinical trials) include IPX203 (novel extended release carbidopa-levodopa capsules), ND062 (carbidopa and levodopa delivered via subcutaneous pump), and ABBV951 (prodrugs of carbidopa and levodopa delivered via subcutaneous pump). Each of these approaches aims to approximate continuous levodopa delivery in an attempt to minimize motor complications.

#### IPX203

IPX203 is a novel oral formulation of carbidopa and levodopa capsules containing different immediate and extended release bead components. It is designed to provide an initial rapid rise in plasma LD followed by prolonged, steady concentrations that extend beyond other currently available oral LD products [221]. In an open-label study [221], 28 patients with motor fluctuations were randomized to 2 weeks treatment with CD-LD IR followed by IPX203 or IPX203 followed by CD-LD IR. Mean dosing frequency at the end of each period was 4.7 for IR CD-LD and 3.1 for IPX203 (*p* < 0.0001). On day 1, after a single dose, LD concentrations were sustained above 50% of *C*_max_ for 4.6 h with IPX203 *versus* 1.5 h with IR CD-LD (*p* < 0.0001). In addition, UPDRS part III scores were significantly more improved with IPX203 compared with IR CD-LD from 3 to 8 h after administration (all *p* < 0.025). PD diaries obtained the last 3 days of each treatment period showed that IPX203 provided a 2.3-h advantage in daily OFF time compared with CD-LD IR (*p* < 0.0001), and daily ON time without troublesome dyskinesia favored IPX203 by 1.9 h (*p* = 0.0001).

In a second open-label study [222], 26 patients with motor fluctuations were randomized to 1 of 3 dosing sequences to receive single doses of CD-LD IR, Rytary, and IPX203. LD plasma concentrations were sustained above 50% *C*_max_ for 1.9 h for CD-LD IR, 3.9 h for Rytary, and 4.7 h for IPX203. Based on hourly MDS-UPDRS part III scores, IPX203 provided significantly greater improvement than CD-LD IR from 3 to 10 h (all *p* ≤ 0.029) and significantly greater improvement compared with Rytary from 5 to 10 h (all *p* ≤ 0.0352) except at 7 h in which the improvement did not reach statistical significance (*p* = 0.0601).

In both of these trials, pharmacokinetic measures demonstrated that IPX203 provides an initial increase in plasma LD concentration similar to CD-LD IR and CD-LD ER, but maintains LD concentrations significantly longer. In addition, IPX203 was demonstrated to reduce peak-trough LD fluctuations compared to CD-LD IR [221, 222]. IPX203 is currently under investigation in a phase 3, randomized, double-blind, double-dummy, active-controlled (IR CD-LD), parallel-group study (IPX203-B16-02, NCT03670953), with patients completing this study also eligible to enroll in a 9-month open-label extension study (IPX203-B16-03, NCT03877510).

#### ND0612

Continuous subcutaneous LD infusion is intended to deliver LD continuously and minimize peak-trough plasma LD fluctuations, thereby reducing motor complications. ND0612 is a continuous subcutaneous infusion of carbidopa and levodopa delivered via mini-pump. In one phase 2 study [223], ND0612 administered as an adjunct to patients’ usual oral regimen maintained steady plasma levodopa concentrations with levodopa troughs eliminated and reduced OFF time when compared to the addition of placebo. Exploratory efficacy analysis showed OFF time as assessed in clinic was reduced by 2.42 h with ND0612 and 0.41 h with placebo. In a subsequent phase 2 study [224], a 24-h infusion of a total daily dose of 720 mg LD and 90 mg carbidopa reduced mean daily OFF time by 2.8 h (*p* = 0.004) and increased mean daily “good” ON time by 3.7 h (*p* < 0.001) compared to baseline, as determined by blinded rater assessments over 8 h. A phase 3 randomized, active-controlled, double-blind, double-dummy, parallel-group trial comparing ND0612 to oral CD-LD IR is currently underway (BouNDless, NCT04006210).

#### ABBV951

ABBV951 is a continuous subcutaneous infusion of carbidopa phosphate and levodopa phosphate (carbidopa and levodopa prodrugs) via pump. Following phase 1 studies investigating pharmacokinetics, dosing, and safety, a phase 3 randomized, double-blind, double-dummy, active-controlled study comparing ABBV951 and CD-LD IR is set to begin enrollment (NCT04380142) [225, 226]. A 52-week, open-label study is also planned (NCT03781167).

## Clinical Management of Motor Complications

In early PD, treatment with CD-LD IR 25–100 mg administered TID (e.g., 7 AM, 12 PM, 5 PM) typically provides relatively sustained relief of motor symptoms through the day with benefit lasting from dose to dose. Once the duration of symptomatic benefit shortens and patients begin to experience OFF episodes, treatment strategies are available to mitigate the impact of these motor fluctuations.

If a patient is experiencing wearing off motor fluctuations on CD-LD IR, a common strategy is to increase the frequency of administration. Many patients will experience resolution of mild wearing off when going from CD-LD IR TID to QID (e.g., 7 AM, 11 AM, 3 PM, 7 PM). If wearing off fluctuations are still present on a QID regimen, further shortening of the interdose interval can be attempted, but such regimens are a challenge for patients and adherence decreases [227, 228]. Therefore, to reduce motor fluctuations in patients who are on CD-LD IR QID, adding an adjunctive medication or switching to CD-LD ER may be advantageous. In our practice, we commonly add one or possibly two adjunctive therapies first, and if that is insufficient, we then favor converting the levodopa regimen to CD-LD ER.

Available adjunctive medications include MAO-B inhibitors (selegiline, rasagiline), mixed selective MAO-B inhibitors and ion channel inhibitors (safinamide, zonisamide [off-label in the USA]), COMT inhibitors (entacapone, opicapone, and tolcapone), an adenosine 2A receptor antagonist (istradefylline), and dopamine agonists (immediate or extended release ropinirole or pramipexole, or transdermal rotigotine). The choice of adjunctive medication typically depends on a combination of patient factors (frequency and severity of OFF episodes, age, and comorbidities) and medication characteristics (efficacy, tolerability, route of elimination and drug interactions, ease of use, accessibility, and cost).

On-demand therapies (subcutaneous and sublingual apomorphine, inhaled levodopa) can also be considered as adjunctive medications for as needed use for patients experiencing OFF episodes during the day. These medications may be particularly helpful in patients with unexpected OFF episodes (as they can be taken on-demand) and for meal associated OFF episodes (as they avoid the GI tract). Subcutaneous apomorphine may also be particularly helpful for morning OFF and deep OFFs during the day. Inhaled levodopa and sublingual apomorphine may be more useful for patients who want to use this type of product multiple times per day while avoiding the need for injections.

For patients who experience OFF overnight, the chief considerations are the severity of the OFF and the time it occurs. For early nighttime OFF, an additional CD-LD IR dose at bedtime may be sufficient. If the OFF occurs later in the night, a bedtime dose of CD-LD ER may be required (potentially enhanced by an adjunct such as opicapone). Another option for nighttime OFF is the addition of a dopamine agonist regimen to provide dopaminergic stimulation through the night. For discrete nighttime OFF episodes, an on-demand medication may be helpful.

For patients who have dyskinesia without OFF time, consideration should be given to reducing or eliminating adjunctive medications or reducing individual LD doses. For patients on CD-LD IR who have both dyskinesia and OFF time, it may be possible to reduce individual levodopa doses and administer them more frequently (fractionation). Alternatively, the patient can be switched from CD-LD IR to CD-LD ER. Both of these strategies are designed to reduce peak-trough fluctuations in serum levodopa concentration. Another important option is the addition of the NMDA receptor antagonist amantadine, which is available in IR and two ER formulations. Amantadine ER capsules (Gocovri™) were demonstrated to significantly reduce both dyskinesia and OFF time in this population.

Cost is also an important factor when considering these therapies, especially for newer medications. Some manufacturers have patient assistance programs that may increase access for some patients.

In patients with motor fluctuations that cannot be adequately controlled with oral medications, consideration can be given to infusion therapies. Outside the USA, CSAI can be added to the patient’s usual regimen to provide a significant reduction in OFF time and increase in ON time without troublesome dyskinesia along with a reduction in oral medications. Alternatively, CLES has been shown to provide a significant reduction in OFF time and increase in ON time without troublesome dyskinesia as a replacement for oral levodopa during the day. Strategically, infusion therapies (those that are currently available and those that are in development) can be used before a patient requires DBS, as an alternative to DBS, or as an adjunct to DBS. New infusion therapies in development that are less invasive than CLES may increase the use of this type of therapy in the future.

## Conclusion

Since its first use in PD more than 50 years ago, levodopa has remained the most effective medical therapy for the treatment of the motor symptoms of PD. For nearly as long, the motor complications associated with levodopa have been the target of immense study [229]. Over the past 30 years, the armamentarium of medical therapies to optimize the motor control of advancing PD has grown, with medications targeting a variety of pathophysiological targets in brain systems underlying these complications now available, and more on the horizon. Navigating the options discussed in this review requires careful evaluation of each individual patient, including the symptoms that most affect their quality of life and factors that affect their susceptibility to adverse effects. Involving patients in shared decision-making regarding the management of evolving motor complications is important as clinicians guide patients through advancing disease [230].

## Electronic supplementary material

ESM 1 (PDF 1224 kb)

## References

[1] Postuma RB, Berg D, Stern M (2015). MDS clinical diagnostic criteria for Parkinson’s disease. Mov Disord.

[2] Chou KL, Stacy M, Simuni T, Miyasaki J, Oertel WH, Sethi K, Fernandez HH, Stocchi F (2018). The spectrum of “off” in Parkinson’s disease: What have we learned over 40 years?. Parkinsonism Relat Disord.

[3] Jankovic J (2005). Motor fluctuations and dyskinesias in Parkinson’s disease: clinical manifestations. Mov Disord.

[4] Hauser RA, Friedlander J, Zesiewicz TA, Adler CH, Seeberger LC, O’Brien CF, Molho ES, Factor SA (2000). A home diary to assess functional status in patients with Parkinson’s disease with motor fluctuations and dyskinesia. Clin Neuropharmacol.

[5] Aquino CC, Fox SH (2015). Clinical spectrum of levodopa-induced complications. Mov Disord.

[6] Rascol O, Brooks DJ, Korczyn AD, De Deyn PP, Clarke CE, Lang AE (2000). A five-year study of the incidence of dyskinesia in patients with early Parkinson’s disease who were treated with ropinirole or levodopa. N Engl J Med.

[7] Marconi R, Lefebvre-Caparros D, Bonnet AM, Vidailhet M, Dubois B, Agid Y (1994). Levodopa-induced dyskinesias in Parkinson’s disease phenomenology and pathophysiology. Mov Disord.

[8] Muenter MD, Sharpless NS, Tyce GM, Darley FL (1977). Patterns of dystonia (“I-D-I” and “D-I-D-”) in response to l-dopa therapy for Parkinson’s disease. Mayo Clin Proc.

[9] Grötzsch H, Sztajzel R, Burkhard PR (2007). Levodopa-induced ocular dyskinesia in Parkinson’s disease. Eur J Neurol.

[10] LeWitt PA (1998). Conjugate eye deviations as dyskinesias induced by levodopa in Parkinson’s disease. Mov Disord.

[11] Rice JE, Antic R, Thompson PD (2002). Disordered respiration as a levodopa-induced dyskinesia in Parkinson’s disease. Mov Disord.

[12] Carecchio M, Collini A, Comi C, Cantello R, Bhatia KP, Monaco F (2010). Levodopa-induced belly dancer’s dyskinesias in Parkinson’s disease: report of one case. Mov Disord.

[13] Fernandez HH, Friedman JH (1999). Punding on L-dopa. Mov Disord.

[14] Fasano A, Ricciardi L, Pettorruso M, Bentivoglio AR (2011). Management of punding in Parkinson’s disease: an open-label prospective study. J Neurol.

[15] Kashihara K, Imamura T (2008). Amantadine may reverse punding in Parkinson’s disease--observation in a patient. Mov Disord.

[16] Aquino CC, Celso de Castro P, Doná F, Medeiros L, Silva SMCA, Borges V, Ferraz HB (2013). Reduction in Parkinson’s disease therapy improved punding but not feeling of presence. J Neuropsychiatr Clin Neurosci.

[17] Silveira-Moriyama L, Evans AH, Katzenschlager R, Lees AJ (2006). Punding and dyskinesias. Mov Disord.

[18] Yoon WT (2018). Comparison of dystonia between Parkinson’s disease and atypical parkinsonism: The clinical usefulness of dystonia distribution and characteristics in the differential diagnosis of parkinsonism. Neurol Neurochir Pol.

[19] Stefanova N, Bücke P, Duerr S, Wenning GK (2009). Multiple system atrophy: an update. Lancet Neurol.

[20] Hely MA, Reid WGJ, Adena MA, Halliday GM, Morris JGL (2008). The Sydney multicenter study of Parkinson’s disease: the inevitability of dementia at 20 years. Mov Disord.

[21] Ahlskog JE, Muenter MD (2001). Frequency of levodopa-related dyskinesias and motor fluctuations as estimated from the cumulative literature. Mov Disord.

[22] Marsden CD, Parkes JD (1977). Success and problems of long-term levodopa therapy in Parkinson’s disease. Lancet.

[23] Stocchi F, Antonini A, Barone P (2014). Early DEtection of wEaring off in Parkinson disease: the DEEP study. Parkinsonism Relat Disord.

[24] Fahn S, Oakes D, Shoulson I, Kieburtz K, Rudolph A, Lang A, Olanow CW, Tanner C, Marek K, Parkinson Study Group (2004). Levodopa and the progression of Parkinson’s disease. N Engl J Med.

[25] Van Gerpen JA, Kumar N, Bower JH, Weigand S, Ahlskog JE (2006). Levodopa-associated dyskinesia risk among Parkinson disease patients in Olmsted County, Minnesota, 1976-1990. Arch Neurol.

[26] Kelly MJ, Lawton MA, Baig F, Ruffmann C, Barber TR, Lo C, Klein JC, Ben-Shlomo Y, Hu MT (2019). Predictors of motor complications in early Parkinson’s disease: A prospective cohort study. Mov Disord.

[27] Hauser RA, McDermott MP, Messing S (2006). Factors associated with the development of motor fluctuations and dyskinesias in Parkinson disease. Arch Neurol.

[28] Warren Olanow C, Kieburtz K, Rascol O, Poewe W, Schapira AH, Emre M, Nissinen H, Leinonen M, Stocchi F, Stalevo Reduction in Dyskinesia Evaluation in Parkinson’s Disease (STRIDE-PD) Investigators (2013). Factors predictive of the development of Levodopa-induced dyskinesia and wearing-off in Parkinson’s disease. Mov Disord.

[29] Mizuno Y, Shimoda S, Origasa H (2018). Long-term treatment of Parkinson’s disease with levodopa and other adjunctive drugs. J Neural Transm.

[30] Chaudhuri KR, Jenner P, Antonini A (2019). Should there be less emphasis on levodopa-induced dyskinesia in Parkinson’s disease?. Mov Disord.

[31] Cenci MA, Riggare S, Pahwa R, Eidelberg D, Hauser RA (2020). Dyskinesia matters. Mov Disord.

[32] Hauser RA, Rascol O, Korczyn AD, Jon Stoessl A, Watts RL, Poewe W, De Deyn PP, Lang AE (2007). Ten-year follow-up of Parkinson’s disease patients randomized to initial therapy with ropinirole or levodopa. Mov Disord.

[33] Parkinson Study Group CALM Cohort Investigators (2009). Long-term effect of initiating pramipexole vs levodopa in early Parkinson disease. Arch Neurol.

[34] Parkinson Study Group (2000). Pramipexole vs levodopa as initial treatment for Parkinson disease: A randomized controlled trial. Parkinson Study Group. JAMA.

[35] Holloway RG, Shoulson I, Fahn S (2004). Pramipexole vs levodopa as initial treatment for Parkinson disease: a 4-year randomized controlled trial. Arch Neurol.

[36] PD MED Collaborative Group (2014). Long-term effectiveness of dopamine agonists and monoamine oxidase B inhibitors compared with levodopa as initial treatment for Parkinson’s disease (PD MED): a large, open-label, pragmatic randomised trial. Lancet.

[37] Verschuur CVM, Suwijn SR, Boel JA (2019). Randomized Delayed-Start Trial of Levodopa in Parkinson’s Disease. N Engl J Med.

[38] Cilia R, Akpalu A, Sarfo FS (2014). The modern pre-levodopa era of Parkinson’s disease: insights into motor complications from sub-Saharan Africa. Brain.

[39] Olanow CW, Obeso JA, Stocchi F (2006). Drug insight: Continuous dopaminergic stimulation in the treatment of Parkinson’s disease. Nat Clin Pract Neurol.

[40] Ray Chaudhuri K, Poewe W, Brooks D (2018). Motor and Nonmotor Complications of Levodopa: Phenomenology, Risk Factors, and Imaging Features. Mov Disord.

[41] Picconi B, Hernández LF, Obeso JA, Calabresi P (2018). Motor complications in Parkinson’s disease: Striatal molecular and electrophysiological mechanisms of dyskinesias. Mov Disord.

[42] Olanow CW, Kieburtz K, Odin P (2014). Continuous intrajejunal infusion of levodopa-carbidopa intestinal gel for patients with advanced Parkinson’s disease: a randomised, controlled, double-blind, double-dummy study. Lancet Neurol.

[43] Antonini A, Nitu B (2018). Apomorphine and levodopa infusion for motor fluctuations and dyskinesia in advanced Parkinson disease. J Neural Transm.

[44] Katzenschlager R, Poewe W, Rascol O (2018). Apomorphine subcutaneous infusion in patients with Parkinson’s disease with persistent motor fluctuations (TOLEDO): a multicentre, double-blind, randomised, placebo-controlled trial. Lancet Neurol.

[45] Stocchi F, Rascol O, Kieburtz K, Poewe W, Jankovic J, Tolosa E, Barone P, Lang AE, Olanow CW (2010). Initiating levodopa/carbidopa therapy with and without entacapone in early Parkinson disease: the STRIDE-PD study. Ann Neurol.

[46] van Wamelen DJ, Grigoriou S, Chaudhuri KR, Odin P (2018). Continuous Drug Delivery Aiming Continuous Dopaminergic Stimulation in Parkinson’s Disease. J Parkinsons Dis.

[47] Freitas ME, Ruiz-Lopez M, Fox SH (2016). Novel Levodopa Formulations for Parkinson’s Disease. CNS Drugs.

[48] Hsu A, Yao H-M, Gupta S, Modi NB (2015). Comparison of the pharmacokinetics of an oral extended-release capsule formulation of carbidopa-levodopa (IPX066) with immediate-release carbidopa-levodopa (Sinemet(®)), sustained-release carbidopa-levodopa (Sinemet(®) CR), and carbidopa-levodopa-entacapone (Stalevo(®)). J Clin Pharmacol.

[49] Hauser RA, Ellenbogen AL, Metman LV, Hsu A, O’Connell MJ, Modi NB, Yao H-M, Kell SH, Gupta SK (2011). Crossover comparison of IPX066 and a standard levodopa formulation in advanced Parkinson’s disease. Mov Disord.

[50] Hauser RA, Hsu A, Kell S, Espay AJ, Sethi K, Stacy M, Ondo W, O’Connell M, Gupta S, IPX066 ADVANCE-PD investigators (2013). Extended-release carbidopa-levodopa (IPX066) compared with immediate-release carbidopa-levodopa in patients with Parkinson’s disease and motor fluctuations: a phase 3 randomised, double-blind trial. Lancet Neurol.

[51] Stocchi F, Hsu A, Khanna S, Ellenbogen A, Mahler A, Liang G, Dillmann U, Rubens R, Kell S, Gupta S (2014). Comparison of IPX066 with carbidopa-levodopa plus entacapone in advanced PD patients. Parkinsonism Relat Disord.

[52] Yao H-M, Hsu A, Gupta S, Modi NB (2016). Clinical Pharmacokinetics of IPX066: Evaluation of Dose Proportionality and Effect of Food in Healthy Volunteers. Clin Neuropharmacol.

[53] Rytary (carbidopa and levodopa extended-release capsules) [package insert on the internet]. Hayward (CA): Impax Pharmaceuticals Inc. 2015 [cited 2020 Jan 20]. Available from: https://www.accessdata.fda.gov/drugsatfda_docs/label/2015/203312s000lbl.pdf

[54] Morgan JC, Dhall R, Rubens R, Khanna S, Gupta S (2018). Dosing Patterns during Conversion to IPX066, Extended-Release Carbidopa-Levodopa (ER CD-LD), in Parkinson’s Disease with Motor Fluctuations. Parkinsons Dis.

[55] Hauser RA (2015). How to dose carbidopa and levodopa extended release capsules (Rytary). Clin Med J.

[56] Espay AJ, Pagan FL, Walter BL (2017). Optimizing extended-release carbidopa/levodopa in Parkinson disease: Consensus on conversion from standard therapy. Neurol Clin Pract.

[57] Pahwa R, Factor SA, Lyons KE (2006). Practice Parameter: treatment of Parkinson disease with motor fluctuations and dyskinesia (an evidence-based review): report of the Quality Standards Subcommittee of the American Academy of Neurology. Neurology.

[58] Jankovic J, Schwartz K, Vander Linden C (1989). Comparison of Sinemet CR4 and standard Sinemet: double blind and long-term open trial in parkinsonian patients with fluctuations. Mov Disord.

[59] Hutton JT, Morris JL, Román GC, Imke SC, Elias JW (1988). Treatment of chronic Parkinson’s disease with controlled-release carbidopa/levodopa. Arch Neurol.

[60] Ahlskog JE, Muenter MD, McManis PG, Bell GN, Bailey PA (1988). Controlled-release Sinemet (CR-4): a double-blind crossover study in patients with fluctuating Parkinson’s disease. Mayo Clin Proc.

[61] Lieberman A, Gopinathan G, Miller E, Neophytides A, Baumann G, Chin L (1990). Randomized double-blind cross-over study of Sinemet-controlled release (CR4 50/200) versus Sinemet 25/100 in Parkinson’s disease. Eur Neurol.

[62] Sinemet CR (carbidopa-levodopa) controlled-release tablets [package insert on the internet]. Whitehouse Station (NJ): Merck and Co, Inc. 1996 [2020 Mar 2]. Available from: https://www.accessdata.fda.gov/drugsatfda_docs/label/2008/019856s025lbl.pdf

[63] Lipp MM, Batycky R, Moore J, Leinonen M, Freed MI (2016). Preclinical and clinical assessment of inhaled levodopa for OFF episodes in Parkinson’s disease. Sci Transl Med.

[64] LeWitt PA, Hauser RA, Grosset DG (2016). A randomized trial of inhaled levodopa (CVT-301) for motor fluctuations in Parkinson’s disease. Mov Disord.

[65] LeWitt PA, Hauser RA, Pahwa R (2019). Safety and efficacy of CVT-301 (levodopa inhalation powder) on motor function during off periods in patients with Parkinson’s disease: a randomised, double-blind, placebo-controlled phase 3 trial. Lancet Neurol.

[66] Hauser RA, Isaacson SH, Ellenbogen A, Safirstein BE, Truong DD, Komjathy SF, Kegler-Ebo DM, Zhao P, Oh C (2019). Orally inhaled levodopa (CVT-301) for early morning OFF periods in Parkinson’s disease. Parkinsonism Relat Disord.

[67] LeWitt PA, Pahwa R, Sedkov A, Corbin A, Batycky R, Murck H (2018). Pulmonary Safety and Tolerability of Inhaled Levodopa (CVT-301) Administered to Patients with Parkinson’s Disease. J Aerosol Med Pulm Drug Deliv.

[68] Grosset DG, Dhall R, Gurevich T, Kassubek J, Poewe WH, Rascol O, Rudzinska M, Cormier J, Sedkov A, Oh C (2020). Inhaled levodopa in Parkinson’s disease patients with OFF periods: A randomized 12-month pulmonary safety study. Parkinsonism Relat Disord.

[69] Inbrija (Levodopa Inhalation Powder) [package insert on the internet]. Ardsley (NY): Acorda Therapeutics, Inc. 2018 [cited 2020 Jan 12]. Available from: https://www.accessdata.fda.gov/drugsatfda_docs/label/2018/209184s000lbl.pdf

[70] Fox SH, Katzenschlager R, Lim S-Y, Barton B, de Bie RMA, Seppi K, Coelho M, Sampaio C, Movement Disorder Society Evidence-Based Medicine Committee (2018). International Parkinson and movement disorder society evidence-based medicine review: Update on treatments for the motor symptoms of Parkinson’s disease. Mov Disord.

[71] Rascol O, Lees AJ, Senard JM, Pirtosek Z, Montastruc JL, Fuell D (1996). Ropinirole in the treatment of levodopa-induced motor fluctuations in patients with Parkinson’s disease. Clin Neuropharmacol.

[72] Lieberman A, Olanow CW, Sethi K, Swanson P, Waters CH, Fahn S, Hurtig H, Yahr M (1998). A multicenter trial of ropinirole as adjunct treatment for Parkinson’s disease. Ropinirole Study Group. Neurology.

[73] Pahwa R, Stacy MA, Factor SA, Lyons KE, Stocchi F, Hersh BP, Elmer LW, Truong DD, Earl NL, EASE-PD Adjunct Study Investigators (2007). Ropinirole 24-hour prolonged release: randomized, controlled study in advanced Parkinson disease. Neurology.

[74] Requip (ropinirole) oral tablets [package insert on the internet]. Research Triangle Park (NC): GlaxoSmithKline, LLC. 2014 [cited 2020 Mar 20]. Available from https://www.accessdata.fda.gov/drugsatfda_docs/label/2014/020658s024s026s027s030s032lbl.pdf

[75] Requip XL (ropinirole) extended-release tablets [package insert on the internet]. Research Triangle Park (NC). 2014 [cited 2020 Mar 20]. Available from: https://www.accessdata.fda.gov/drugsatfda_docs/label/2014/022008s003s004s007s008lbledt.pdf

[76] Lieberman A, Ranhosky A, Korts D (1997). Clinical evaluation of pramipexole in advanced Parkinson’s disease: results of a double-blind, placebo-controlled, parallel-group study. Neurology.

[77] Möller JC, Oertel WH, Köster J, Pezzoli G, Provinciali L (2005). Long-term efficacy and safety of pramipexole in advanced Parkinson’s disease: results from a European multicenter trial. Mov Disord.

[78] Schapira AHV, Barone P, Hauser RA, Mizuno Y, Rascol O, Busse M, Salin L, Juhel N, Poewe W, Pramipexole ER Studies Group (2011). Extended-release pramipexole in advanced Parkinson disease: a randomized controlled trial. Neurology.

[79] Mirapex (pramipexole dihydrochloride) tablets [package insert on the internet]. Ridgefield CT: Boehringer Ingelheim Pharmaceuticals, Inc. 2007 [cited 2020 Mar 20]. Available from: https://www.accessdata.fda.gov/drugsatfda_docs/label/2008/020667s014s017s018lbl.pdf

[80] Mirapex ER (Pramipexole dihydrochloride Extended-release) Oral Tablet package insert on the internet]. Ridgefield (CT): Boehringer Ingelheim Pharmaceuticals, Inc. 2014 [cited 2020 Mar 20]. Available from: https://www.accessdata.fda.gov/drugsatfda_docs/label/2014/022421s003lbl.pdf

[81] LeWitt PA, Lyons KE, Pahwa R, SP 650 Study Group (2007). Advanced Parkinson disease treated with rotigotine transdermal system: PREFER Study. Neurology.

[82] Poewe WH, Rascol O, Quinn N, Tolosa E, Oertel WH, Martignoni E, Rupp M, Boroojerdi B, SP 515 Investigators (2007). Efficacy of pramipexole and transdermal rotigotine in advanced Parkinson’s disease: a double-blind, double-dummy, randomised controlled trial. Lancet Neurol.

[83] LeWitt PA, Boroojerdi B, Surmann E, Poewe W, SP716 Study Group, SP715 Study Group (2013). Rotigotine transdermal system for long-term treatment of patients with advanced Parkinson’s disease: results of two open-label extension studies, CLEOPATRA-PD and PREFER. J Neural Transm.

[84] Neupro (rotigotine) film label [package insert on the internet]. Smyrna (GA): UCB, Inc. 2012 [cited 2020 Mar 20]. Available from: https://www.accessdata.fda.gov/drugsatfda_docs/label/2012/021829s001lbl.pdf

[85] Carbone F, Djamshidian A, Seppi K, Poewe W (2019). Apomorphine for Parkinson’s Disease: Efficacy and Safety of Current and New Formulations. CNS Drugs.

[86] Kempster PA, Frankel JP, Stern GM, Lees AJ (1990). Comparison of motor response to apomorphine and levodopa in Parkinson’s disease. J Neurol Neurosurg Psychiatry.

[87] Merello M, Pikielny R, Cammarota A, Leiguarda R (1997). Comparison of subcutaneous apomorphine versus dispersible madopar latency and effect duration in Parkinson’s disease patients: a double-blind single-dose study. Clin Neuropharmacol.

[88] Stibe CM, Lees AJ, Kempster PA, Stern GM (1988). Subcutaneous apomorphine in parkinsonian on-off oscillations. Lancet.

[89] Frankel JP, Lees AJ, Kempster PA, Stern GM (1990). Subcutaneous apomorphine in the treatment of Parkinson’s disease. J Neurol Neurosurg Psychiatry.

[90] Hughes AJ, Bishop S, Kleedorfer B, Turjanski N, Fernandez W, Lees AJ, Stern GM (1993). Subcutaneous apomorphine in Parkinson’s disease: response to chronic administration for up to five years. Mov Disord.

[91] Dewey RB, Hutton JT, LeWitt PA, Factor SA (2001). A randomized, double-blind, placebo-controlled trial of subcutaneously injected apomorphine for parkinsonian off-state events. Arch Neurol.

[92] Pfeiffer RF, Gutmann L, Hull KL, Bottini PB, Sherry JH, APO302 Study Investigators (2007). Continued efficacy and safety of subcutaneous apomorphine in patients with advanced Parkinson’s disease. Parkinsonism Relat Disord.

[93] Pahwa R, Koller WC, Trosch RM, Sherry JH, APO303 Study Investigators (2007). Subcutaneous apomorphine in patients with advanced Parkinson’s disease: a dose-escalation study with randomized, double-blind, placebo-controlled crossover evaluation of a single dose. J Neurol Sci.

[94] Isaacson S, Lew M, Ondo W, Hubble J, Clinch T, Pagan F (2017). Apomorphine Subcutaneous Injection for the Management of Morning Akinesia in Parkinson’s Disease. Mov Disord Clin Pract.

[95] Hauser RA, Isaacson S, Clinch T, Tigan/Apokyn Study Investigators (2014). Randomized, placebo-controlled trial of trimethobenzamide to control nausea and vomiting during initiation and continued treatment with subcutaneous apomorphine injection. Parkinsonism Relat Disord.

[96] Bhidayasiri R, Chaudhuri KR, LeWitt P, Martin A, Boonpang K, van Laar T (2015). Effective delivery of apomorphine in the management of Parkinson disease: practical considerations for clinicians and Parkinson nurses. Clin Neuropharmacol.

[97] APOKYN (apomorphine hydrochloride, USP) [package insert on the internet]. Louisville (KY): US WorldMeds, LLC, 2014. [cited 2020 Mar 20]. Available from: https://www.apokyn.com/sites/all/themes/apokyn/content/resources/Apokyn_PI.pdf

[98] Olanow CW, Factor SA, Espay AJ, et al (2019) Apomorphine sublingual film for off episodes in Parkinson’s disease: a randomised, double-blind, placebo-controlled phase 3 study. Lancet Neurol 10.1016/S1474-4422(19)30396-5 10.1016/S1474-4422(19)30396-5 31818699

[99] Hauser RA, Olanow CW, Dzyngel B, Bilbault T, Shill H, Isaacson S, Dubow J, Agro A (2016). Sublingual apomorphine (APL-130277) for the acute conversion of OFF to ON in Parkinson’s disease. Mov Disord.

[100] Kynmobi (apomorphine hydrochloride) sublingual film [package insert on the internet]. Marlborough (MA): Sunovion Pharmaceuticals Inc. 2020 [cited 2020 Jun 16]. Available from: accessdata.fda.gov/drugsatfda_docs/label/2020/210875lbl.pdf

[101] Antonini A, Tolosa E, Mizuno Y, Yamamoto M, Poewe WH (2009). A reassessment of risks and benefits of dopamine agonists in Parkinson’s disease. Lancet Neurol.

[102] Guttman M, Léger G, Reches A, Evans A, Kuwabara H, Cedarbaum JM, Gjedde A (1993). Administration of the new COMT inhibitor OR-611 increases striatal uptake of fluorodopa. Mov Disord.

[103] Dingemanse J, Jorga K, Zürcher G, Schmitt M, Sedek G, Da Prada M, Van Brummelen P (1995). Pharmacokinetic-pharmacodynamic interaction between the COMT inhibitor tolcapone and single-dose levodopa. Br J Clin Pharmacol.

[104] Kaakkola S (2000). Clinical pharmacology, therapeutic use and potential of COMT inhibitors in Parkinson’s disease. Drugs.

[105] Keränen T, Gordin A, Harjola VP, Karlsson M, Korpela K, Pentikäinen PJ, Rita H, Seppälä L, Wikberg T (1993). The effect of catechol-O-methyl transferase inhibition by entacapone on the pharmacokinetics and metabolism of levodopa in healthy volunteers. Clin Neuropharmacol.

[106] Sêdek G, Jorga K, Schmitt M, Burns RS, Leese P (1997). Effect of tolcapone on plasma levodopa concentrations after coadministration with levodopa/carbidopa to healthy volunteers. Clin Neuropharmacol.

[107] (1997) Entacapone improves motor fluctuations in levodopa-treated Parkinson’s disease patients. Parkinson Study Group. Ann Neurol 42:747–755 10.1002/ana.410420511 9392574

[108] Poewe WH, Deuschl G, Gordin A, Kultalahti E-R, Leinonen M, Celomen Study Group (2002). Efficacy and safety of entacapone in Parkinson’s disease patients with suboptimal levodopa response: a 6-month randomized placebo-controlled double-blind study in Germany and Austria (Celomen study). Acta Neurol Scand.

[109] Rinne UK, Larsen JP, Siden A, Worm-Petersen J (1998). Entacapone enhances the response to levodopa in parkinsonian patients with motor fluctuations. Nomecomt Study Group. Neurology.

[110] Brusa L, Bassi A, Lunardi G, Fedele E, Peppe A, Stefani A, Pasqualetti P, Stanzione P, Pierantozzi M (2004). Delayed administration may improve entacapone effects in parkinsonian patients non-responding to the drug. Eur J Neurol.

[111] Comtan (entacapone) tablets [package insert on the internet]. East Hanover (NJ): Novartis Pharmaceuticals Corporation. 2014 [cited 2020 Feb 10]. Available from: https://www.accessdata.fda.gov/drugsatfda_docs/label/2014/020796s016lbl.pdf

[112] Rocha J-F, Falcão A, Santos A, Pinto R, Lopes N, Nunes T, Wright LC, Vaz-da-Silva M, Soares-da-Silva P (2014). Effect of opicapone and entacapone upon levodopa pharmacokinetics during three daily levodopa administrations. Eur J Clin Pharmacol.

[113] Ferreira JJ, Lees A, Rocha J-F, Poewe W, Rascol O, Soares-da-Silva P, Bi-Park 1 investigators (2016). Opicapone as an adjunct to levodopa in patients with Parkinson’s disease and end-of-dose motor fluctuations: a randomised, double-blind, controlled trial. Lancet Neurol.

[114] Lees AJ, Ferreira J, Rascol O, Poewe W, Rocha J-F, McCrory M, Soares-da-Silva P, BIPARK-2 Study Investigators (2017). Opicapone as Adjunct to Levodopa Therapy in Patients With Parkinson Disease and Motor Fluctuations: A Randomized Clinical Trial. JAMA Neurol.

[115] Fabbri M, Ferreira JJ, Lees A, Stocchi F, Poewe W, Tolosa E, Rascol O (2018). Opicapone for the treatment of Parkinson’s disease: A review of a new licensed medicine. Mov Disord.

[116] Lees A, Ferreira JJ, Rocha J-F, Rascol O, Poewe W, Gama H, Soares-da-Silva P (2019). Safety Profile of Opicapone in the Management of Parkinson’s Disease. J Parkinsons Dis.

[117] Ogentys (opicapone) capsules [package insert on the internet]. San Diego (CA). Neurocrine Biosciences, Inc. 2020 (2020 Jun 16). Available from: https://www.accessdata.fda.gov/drugsatfda_docs/label/2020/212489s000lbl.pdf

[118] Rajput AH, Martin W, Saint-Hilaire MH, Dorflinger E, Pedder S (1997). Tolcapone improves motor function in parkinsonian patients with the “wearing-off” phenomenon: a double-blind, placebo-controlled, multicenter trial. Neurology.

[119] Baas H, Beiske AG, Ghika J, Jackson M, Oertel WH, Poewe W, Ransmayr G (1997). Catechol-O-methyltransferase inhibition with tolcapone reduces the “wearing off” phenomenon and levodopa requirements in fluctuating parkinsonian patients. J Neurol Neurosurg Psychiatry.

[120] Adler CH, Singer C, O’Brien C, Hauser RA, Lew MF, Marek KL, Dorflinger E, Pedder S, Deptula D, Yoo K (1998). Randomized, placebo-controlled study of tolcapone in patients with fluctuating Parkinson disease treated with levodopa-carbidopa. Tolcapone Fluctuator Study Group III. Arch Neurol.

[121] Olanow CW (2000). Tolcapone and hepatotoxic effects. Tasmar Advisory Panel. Arch Neurol.

[122] Olanow CW, Watkins PB (2007). Tolcapone: an efficacy and safety review (2007). Clin Neuropharmacol.

[123] Eggert K, Oertel WH, Lees AJ, German Competence Network on Parkinson’s disease (2014) Safety and efficacy of tolcapone in the long-term use in Parkinson disease: an observational study. Clin Neuropharmacol 37:1–5 10.1097/WNF.0000000000000008 24434524

[124] Tasmar (tolcapone) [package insert on the internet]. Bridgewater (NJ). Humacao (PR): Legacy Pharmaceuticals Puerto Rico, LLC. 2013 [cited: 2020 Jan 15]. Available from: https://www.accessdata.fda.gov/drugsatfda_docs/label/2013/020697s004lbl.pdf

[125] Entacapone to Tolcapone Switch Study Investigators (2007). Entacapone to tolcapone switch: Multicenter double-blind, randomized, active-controlled trial in advanced Parkinson’s disease. Mov Disord.

[126] Onofrj M, Thomas A, Iacono D, Di Iorio A, Bonanni L (2001). Switch-over from tolcapone to entacapone in severe Parkinson’s disease patients. Eur Neurol.

[127] Lees AJ (2008). Evidence-based efficacy comparison of tolcapone and entacapone as adjunctive therapy in Parkinson’s disease. CNS Neurosci Ther.

[128] Ferreira JJ, Lees AJ, Poewe W, Rascol O, Rocha J-F, Keller B, Soares-da-Silva P (2018). Effectiveness of opicapone and switching from entacapone in fluctuating Parkinson disease. Neurology.

[129] Brooks DJ, Agid Y, Eggert K, Widner H, Ostergaard K, Holopainen A, TC-INIT Study Group (2005). Treatment of end-of-dose wearing-off in parkinson’s disease: stalevo (levodopa/carbidopa/entacapone) and levodopa/DDCI given in combination with Comtess/Comtan (entacapone) provide equivalent improvements in symptom control superior to that of traditional levodopa/DDCI treatment. Eur Neurol.

[130] Lyons KE, Pahwa R (2006). Conversion from sustained release carbidopa/levodopa to carbidopa/levodopa/entacapone (stalevo) in Parkinson disease patients. Clin Neuropharmacol.

[131] Myllylä V, Haapaniemi T, Kaakkola S (2006). Patient satisfaction with switching to Stalevo: an open-label evaluation in PD patients experiencing wearing-off (Simcom Study). Acta Neurol Scand.

[132] Stalevo (carbidopa, levodopa, and entacapone) tablets, [package insert on the internet]. Espoo (Finland): Orion Pharma. 2003 [cited 2020 Mar 10]. Available from: https://www.accessdata.fda.gov/drugsatfda_docs/label/2014/021485s028s029lbl.pdf

[133] Chen JJ, Swope DM, Dashtipour K (2007). Comprehensive review of rasagiline, a second-generation monoamine oxidase inhibitor, for the treatment of Parkinson’s disease. Clin Ther.

[134] deMarcaida JA, Schwid SR, White WB, Blindauer K, Fahn S, Kieburtz K, Stern M, Shoulson I, Parkinson Study Group TEMPO, PRESTO Tyramine Substudy Investigators and Coordinators (2006). Effects of tyramine administration in Parkinson’s disease patients treated with selective MAO-B inhibitor rasagiline. Mov Disord.

[135] Oreland L, Jossan SS, Hartvig P, Aquilonius SM, Långström B (1990). Turnover of monoamine oxidase B (MAO-B) in pig brain by positron emission tomography using 11C-L-deprenyl. J Neural Transm Suppl.

[136] Mahmood I (1997). Clinical pharmacokinetics and pharmacodynamics of selegiline. An update. Clin Pharmacokinet.

[137] Freedman NMT, Mishani E, Krausz Y, Weininger J, Lester H, Blaugrund E, Ehrlich D, Chisin R (2005). In vivo measurement of brain monoamine oxidase B occupancy by rasagiline, using (11)C-l-deprenyl and PET. J Nucl Med.

[138] Golbe LI, Lieberman AN, Muenter MD, Ahlskog JE, Gopinathan G, Neophytides AN, Foo SH, Duvoisin RC (1988). Deprenyl in the treatment of symptom fluctuations in advanced Parkinson’s disease. Clin Neuropharmacol.

[139] Golbe LI, Duvoisin RC (1987). Double-blind trial of R-(-)-deprenyl for the “on-off” effect complicating Parkinson’s disease. J Neural Transm Suppl.

[140] Waters CH, Sethi KD, Hauser RA, Molho E, Bertoni JM, Zydis Selegiline Study Group (2004). Zydis selegiline reduces off time in Parkinson’s disease patients with motor fluctuations: a 3-month, randomized, placebo-controlled study. Mov Disord.

[141] Ondo WG, Sethi KD, Kricorian G (2007). Selegiline orally disintegrating tablets in patients with Parkinson disease and “wearing off” symptoms. Clin Neuropharmacol.

[142] Lew MF, Pahwa R, Leehey M, Bertoni J, Kricorian G, Zydis Selegiline Study Group (2007). Safety and efficacy of newly formulated selegiline orally disintegrating tablets as an adjunct to levodopa in the management of “off” episodes in patients with Parkinson’s disease. Curr Med Res Opin.

[143] Eldepryl (selegiline hydrochloride) Capsules [package insert on the internet]. Tampa (FL): Somerset Pharmaceuticals, Inc. 2008 [cited 2020 Mar 15]. Available from: https://www.accessdata.fda.gov/drugsatfda_docs/label/2008/020647s006s007lbl.pdf

[144] Zelapar (selegiline hydrochloride) orally disintegrating tablets. Swindon (UK): Cardinal Health, Inc. 2008 [cited 2020 Mar 15]. Available from: https://www.accessdata.fda.gov/drugsatfda_docs/label/2008/021479s003s004lbl.pdf

[145] Rascol O, Brooks DJ, Melamed E, Oertel W, Poewe W, Stocchi F, Tolosa E, LARGO study group (2005). Rasagiline as an adjunct to levodopa in patients with Parkinson’s disease and motor fluctuations (LARGO, Lasting effect in Adjunct therapy with Rasagiline Given Once daily, study): a randomised, double-blind, parallel-group trial. Lancet.

[146] Parkinson Study Group (2005). A randomized placebo-controlled trial of rasagiline in levodopa-treated patients with Parkinson disease and motor fluctuations: the PRESTO study. Arch Neurol.

[147] Azilect (rasagiline mesylate) Tablet, [package insert on the internet]. North Wales (PA): TEVA Neuroscience, Inc. 2014 [cited 2020 Mar 15]. Available from: https://www.accessdata.fda.gov/drugsatfda_docs/label/2014/021641s016s017lbl.pdf

[148] Müller T, Foley P (2017). Clinical Pharmacokinetics and Pharmacodynamics of Safinamide. Clin Pharmacokinet.

[149] Borgohain R, Szasz J, Stanzione P (2014). Randomized trial of safinamide add-on to levodopa in Parkinson’s disease with motor fluctuations. Mov Disord.

[150] Borgohain R, Szasz J, Stanzione P (2014). Two-year, randomized, controlled study of safinamide as add-on to levodopa in mid to late Parkinson’s disease. Mov Disord.

[151] Schapira AHV, Fox SH, Hauser RA, Jankovic J, Jost WH, Kenney C, Kulisevsky J, Pahwa R, Poewe W, Anand R (2017). Assessment of Safety and Efficacy of Safinamide as a Levodopa Adjunct in Patients With Parkinson Disease and Motor Fluctuations: A Randomized Clinical Trial. JAMA Neurol.

[152] XADAGO (safinamide) tablets [package insert on the internet]. Louisville (KY): US WorldMeds, LLC. 2017 [cited 2020 Mar 15]. Available from: https://www.accessdata.fda.gov/drugsatfda_docs/label/2017/207145lbl.pdf

[153] Murata M, Hasegawa K, Kanazawa I, Fukasaka J, Kochi K, Shimazu R, Japan Zonisamide on PD Study Group (2015). Zonisamide improves wearing-off in Parkinson’s disease: A randomized, double-blind study. Mov Disord.

[154] Murata M, Hasegawa K, Kanazawa I, Japan Zonisamide on PD Study Group (2007). Zonisamide improves motor function in Parkinson disease: a randomized, double-blind study. Neurology.

[155] Murata M, Horiuchi E, Kanazawa I (2001). Zonisamide has beneficial effects on Parkinson’s disease patients. Neurosci Res.

[156] Murata M (2004). Novel therapeutic effects of the anti-convulsant, zonisamide, on Parkinson’s disease. Curr Pharm Des.

[157] Zonegran (zonisamide) capsules [package insert on the internet]. Woodcliff Lake (NJ): Elan Pharma International Ltd. 2009 [cited 2020 Mar 23]. Available from: https://www.accessdata.fda.gov/drugsatfda_docs/label/2009/020789s022s025lbl.pdf

[158] Kase H (2001). New aspects of physiological and pathophysiological functions of adenosine A2A receptor in basal ganglia. Biosci Biotechnol Biochem.

[159] Ochi M, Shiozaki S, Kase H (2004). Adenosine A(2A) receptor-mediated modulation of GABA and glutamate release in the output regions of the basal ganglia in a rodent model of Parkinson’s disease. Neuroscience.

[160] Grondin R, Bédard PJ, Hadj Tahar A, Grégoire L, Mori A, Kase H (1999). Antiparkinsonian effect of a new selective adenosine A2A receptor antagonist in MPTP-treated monkeys. Neurology.

[161] Kanda T, Tashiro T, Kuwana Y, Jenner P (1998). Adenosine A2A receptors modify motor function in MPTP-treated common marmosets. Neuroreport.

[162] Kanda T, Jackson MJ, Smith LA, Pearce RK, Nakamura J, Kase H, Kuwana Y, Jenner P (1998). Adenosine A2A antagonist: a novel antiparkinsonian agent that does not provoke dyskinesia in parkinsonian monkeys. Ann Neurol.

[163] LeWitt PA, Guttman M, Tetrud JW, Tuite PJ, Mori A, Chaikin P, Sussman NM, 6002-US-005 Study Group (2008). Adenosine A2A receptor antagonist istradefylline (KW-6002) reduces “off” time in Parkinson’s disease: a double-blind, randomized, multicenter clinical trial (6002-US-005). Ann Neurol.

[164] Hauser RA, Shulman LM, Trugman JM, Roberts JW, Mori A, Ballerini R, Sussman NM, Istradefylline 6002-US-013 Study Group (2008). Study of istradefylline in patients with Parkinson’s disease on levodopa with motor fluctuations. Mov Disord.

[165] Mizuno Y, Hasegawa K, Kondo T, Kuno S, Yamamoto M, Japanese Istradefylline Study Group (2010). Clinical efficacy of istradefylline (KW-6002) in Parkinson’s disease: a randomized, controlled study. Mov Disord.

[166] Mizuno Y, Kondo T, Japanese Istradefylline Study Group (2013). Adenosine A2A receptor antagonist istradefylline reduces daily OFF time in Parkinson’s disease. Mov Disord.

[167] Takahashi M, Fujita M, Asai N, Saki M, Mori A (2018). Safety and effectiveness of istradefylline in patients with Parkinson’s disease: interim analysis of a post-marketing surveillance study in Japan. Expert Opin Pharmacother.

[168] Istradefylline [package insert on the Internet]. Bedminseter (NJ): Kyowa Kirin, 2019 [cited 2019 Dec 6]. Available from: URL: https://www.nourianzhcp.com/assets/pdf/nourianz-full-prescribing-information.pdf

[169] Fox SH (2013). Non-dopaminergic treatments for motor control in Parkinson’s disease. Drugs.

[170] Engber TM, Papa SM, Boldry RC, Chase TN (1994). NMDA receptor blockade reverses motor response alterations induced by levodopa. Neuroreport.

[171] Verhagen Metman L, Del Dotto P, van den Munckhof P, Fang J, Mouradian MM, Chase TN (1998). Amantadine as treatment for dyskinesias and motor fluctuations in Parkinson’s disease. Neurology.

[172] Metman LV, Del Dotto P, LePoole K, Konitsiotis S, Fang J, Chase TN (1999). Amantadine for levodopa-induced dyskinesias: a 1-year follow-up study. Arch Neurol.

[173] Snow BJ, Macdonald L, Mcauley D, Wallis W (2000). The effect of amantadine on levodopa-induced dyskinesias in Parkinson’s disease: a double-blind, placebo-controlled study. Clin Neuropharmacol.

[174] Luginger E, Wenning GK, Bösch S, Poewe W (2000). Beneficial effects of amantadine on L-dopa-induced dyskinesias in Parkinson’s disease. Mov Disord.

[175] Sawada H, Oeda T, Kuno S, Nomoto M, Yamamoto K, Yamamoto M, Hisanaga K, Kawamura T, Amantadine Study Group (2010). Amantadine for dyskinesias in Parkinson’s disease: a randomized controlled trial. PLoS One.

[176] Ory-Magne F, Corvol J-C, Azulay J-P (2014). Withdrawing amantadine in dyskinetic patients with Parkinson disease: the AMANDYSK trial. Neurology.

[177] Wolf E, Seppi K, Katzenschlager R (2010). Long-term antidyskinetic efficacy of amantadine in Parkinson’s disease. Mov Disord.

[178] Oertel W, Eggert K, Pahwa R (2017). Randomized, placebo-controlled trial of ADS-5102 (amantadine) extended-release capsules for levodopa-induced dyskinesia in Parkinson’s disease (EASE LID 3). Mov Disord.

[179] Pahwa R, Tanner CM, Hauser RA (2017). ADS-5102 (Amantadine) Extended-Release Capsules for Levodopa-Induced Dyskinesia in Parkinson Disease (EASE LID Study): A Randomized Clinical Trial. JAMA Neurol.

[180] Elmer LW, Juncos JL, Singer C, Truong DD, Criswell SR, Parashos S, Felt L, Johnson R, Patni R (2018). Pooled Analyses of Phase III Studies of ADS-5102 (Amantadine) Extended-Release Capsules for Dyskinesia in Parkinson’s Disease. CNS Drugs.

[181] Hauser RA, Kremens DE, Elmer LW, Kreitzman DL, Walsh RR, Johnson R, Howard R, Nguyen JT, Patni R (2019). Prevalence of Dyskinesia and OFF by 30-Minute Intervals Through the Day and Assessment of Daily Episodes of Dyskinesia and OFF: Novel Analyses of Diary Data from Gocovri Pivotal Trials. J Parkinsons Dis.

[182] GOCOVRI (amantadine) extended release capsules [package insert on the internet]. Adamas Pharma, LLC. Emeryville (CA). 2017 [2019 Dec 13]. Available from: https://www.accessdata.fda.gov/drugsatfda_docs/label/2017/208944lbl.pdf

[183] Osmolex ER (amantadine) extended-release tablets [package insert on the internet]. Bridgewater (NJ). Vertical Pharmaceuticals, LLC. 2018 (2019 Dec 13). Available from: https://www.accessdata.fda.gov/drugsatfda_docs/label/2018/209410s000lbl.pdf

[184] deVries T, Dentiste A, Di Lea C, Pichette V, Jacobs D (2019). Effects of Renal Impairment on the Pharmacokinetics of Once-Daily Amantadine Extended-Release Tablets. CNS Drugs.

[185] Arevalo GJ, Gershanik OS (1993). Modulatory effect of clozapine on levodopa response in Parkinson’s disease: a preliminary study. Mov Disord.

[186] Durif F, Vidailhet M, Assal F, Roche C, Bonnet AM, Agid Y (1997). Low-dose clozapine improves dyskinesias in Parkinson’s disease. Neurology.

[187] Bennett JP, Landow ER, Schuh LA (1993). Suppression of dyskinesias in advanced Parkinson’s disease. II. Increasing daily clozapine doses suppress dyskinesias and improve parkinsonism symptoms. Neurology.

[188] Bennett JP, Landow ER, Dietrich S, Schuh LA (1994). Suppression of dyskinesias in advanced Parkinson’s disease: moderate daily clozapine doses provide long-term dyskinesia reduction. Mov Disord.

[189] Pierelli F, Adipietro A, Soldati G, Fattapposta F, Pozzessere G, Scoppetta C (1998). Low dosage clozapine effects on L-dopa induced dyskinesias in parkinsonian patients. Acta Neurol Scand.

[190] Durif F, Debilly B, Galitzky M, Morand D, Viallet F, Borg M, Thobois S, Broussolle E, Rascol O (2004). Clozapine improves dyskinesias in Parkinson disease: a double-blind, placebo-controlled study. Neurology.

[191] Pietz K, Hagell P, Odin P (1998). Subcutaneous apomorphine in late stage Parkinson’s disease: a long term follow up. J Neurol Neurosurg Psychiatry.

[192] Stocchi F, Vacca L, De Pandis MF, Barbato L, Valente M, Ruggieri S (2001). Subcutaneous continuous apomorphine infusion in fluctuating patients with Parkinson’s disease: long-term results. Neurol Sci.

[193] Manson AJ, Turner K, Lees AJ (2002). Apomorphine monotherapy in the treatment of refractory motor complications of Parkinson’s disease: long-term follow-up study of 64 patients. Mov Disord.

[194] García Ruiz PJ, Sesar Ignacio A, Ares Pensado B (2008). Efficacy of long-term continuous subcutaneous apomorphine infusion in advanced Parkinson’s disease with motor fluctuations: a multicenter study. Mov Disord.

[195] Drapier S, Gillioz A-S, Leray E, Péron J, Rouaud T, Marchand A, Vérin M (2012). Apomorphine infusion in advanced Parkinson’s patients with subthalamic stimulation contraindications. Parkinsonism Relat Disord.

[196] Kimber TE, Fang J, Huddy LJ, Thompson PD (2017). Long-term adherence to apomorphine infusion in patients with Parkinson disease: a 10-year observational study. Intern Med J.

[197] Martinez-Martin P, Reddy P, Katzenschlager R (2015). EuroInf: a multicenter comparative observational study of apomorphine and levodopa infusion in Parkinson’s disease. Mov Disord.

[198] Drapier S, Eusebio A, Degos B (2016). Quality of life in Parkinson’s disease improved by apomorphine pump: the OPTIPUMP cohort study. J Neurol.

[199] Borgemeester RWK, Drent M, van Laar T (2016). Motor and non-motor outcomes of continuous apomorphine infusion in 125 Parkinson’s disease patients. Parkinsonism Relat Disord.

[200] Sesar Á, Fernández-Pajarín G, Ares B, Rivas MT, Castro A (2017). Continuous subcutaneous apomorphine infusion in advanced Parkinson’s disease: 10-year experience with 230 patients. J Neurol.

[201] Dafsari HS, Martinez-Martin P, Rizos A (2019). EuroInf 2: Subthalamic stimulation, apomorphine, and levodopa infusion in Parkinson’s disease. Mov Disord.

[202] Borgemeester RWK, Lees AJ, van Laar T (2016). Parkinson’s disease, visual hallucinations and apomorphine: A review of the available evidence. Parkinsonism Relat Disord.

[203] Shoulson I, Glaubiger GA, Chase TN (1975). On-off response. Clinical and biochemical correlations during oral and intravenous levodopa administration in parkinsonian patients. Neurology.

[204] Quinn N, Parkes JD, Marsden CD (1984). Control of on/off phenomenon by continuous intravenous infusion of levodopa. Neurology.

[205] Quinn N, Marsden CD, Parkes JD (1982). Complicated response fluctuations in Parkinson’s disease: response to intravenous infusion of levodopa. Lancet.

[206] Syed N, Murphy J, Zimmerman T, Mark MH, Sage JI (1998). Ten years’ experience with enteral levodopa infusions for motor fluctuations in Parkinson’s disease. Mov Disord.

[207] Kurlan R, Rubin AJ, Miller C, Rivera-Calimlim L, Clarke A, Shoulson I (1986). Duodenal delivery of levodopa for on-off fluctuations in parkinsonism: preliminary observations. Ann Neurol.

[208] Kurlan R, Nutt JG, Woodward WR, Rothfield K, Lichter D, Miller C, Carter JH, Shoulson I (1988). Duodenal and gastric delivery of levodopa in parkinsonism. Ann Neurol.

[209] Nyholm D, Askmark H, Gomes-Trolin C, Knutson T, Lennernäs H, Nyström C, Aquilonius S-M (2003). Optimizing levodopa pharmacokinetics: intestinal infusion versus oral sustained-release tablets. Clin Neuropharmacol.

[210] Nilsson D, Nyholm D, Aquilonius SM (2001). Duodenal levodopa infusion in Parkinson’s disease--long-term experience. Acta Neurol Scand.

[211] Nyholm D, Nilsson Remahl AIM, Dizdar N, Constantinescu R, Holmberg B, Jansson R, Aquilonius S-M, Askmark H (2005). Duodenal levodopa infusion monotherapy vs oral polypharmacy in advanced Parkinson disease. Neurology.

[212] Eggert K, Schrader C, Hahn M, Stamelou M, Rüssmann A, Dengler R, Oertel W, Odin P (2008). Continuous jejunal levodopa infusion in patients with advanced parkinson disease: practical aspects and outcome of motor and non-motor complications. Clin Neuropharmacol.

[213] Antonini A, Isaias IU, Canesi M, Zibetti M, Mancini F, Manfredi L, Dal Fante M, Lopiano L, Pezzoli G (2007). Duodenal levodopa infusion for advanced Parkinson’s disease: 12-month treatment outcome. Mov Disord.

[214] Zibetti M, Merola A, Artusi CA, Rizzi L, Angrisano S, Reggio D, De Angelis C, Rizzone M, Lopiano L (2014). Levodopa/carbidopa intestinal gel infusion in advanced Parkinson’s disease: a 7-year experience. Eur J Neurol.

[215] Zibetti M, Merola A, Ricchi V (2013). Long-term duodenal levodopa infusion in Parkinson’s disease: a 3-year motor and cognitive follow-up study. J Neurol.

[216] Slevin JT, Fernandez HH, Zadikoff C, Hall C, Eaton S, Dubow J, Chatamra K, Benesh J (2015). Long-term safety and maintenance of efficacy of levodopa-carbidopa intestinal gel: an open-label extension of the double-blind pivotal study in advanced Parkinson’s disease patients. J Parkinsons Dis.

[217] Antonini A, Fung VSC, Boyd JT, Slevin JT, Hall C, Chatamra K, Eaton S, Benesh JA (2016). Effect of levodopa-carbidopa intestinal gel on dyskinesia in advanced Parkinson’s disease patients. Mov Disord.

[218] Fernandez HH, Standaert DG, Hauser RA (2015). Levodopa-carbidopa intestinal gel in advanced Parkinson’s disease: final 12-month, open-label results. Mov Disord.

[219] Amjad F, Bhatti D, Davis TL, Oguh O, Pahwa R, Kukreja P, Zamudio J, Metman LV (2019). Current Practices for Outpatient Initiation of Levodopa-Carbidopa Intestinal Gel for Management of Advanced Parkinson’s Disease in the United States. Adv Ther.

[220] DUOPA (carbidopa and levodopa enteral suspecion) [package insert on the internet]. North Chicago (IL). AbbVie Inc. 2019 [2020 Jan 20]. Available from: https://www.rxabbvie.com/pdf/duopa_pi.pdf

[221] Modi NB, Mittur A, Dinh P, Rubens R, Gupta S (2019). Pharmacodynamics, Efficacy, and Safety of IPX203 in Parkinson Disease Patients With Motor Fluctuations. Clin Neuropharmacol.

[222] Modi NB, Mittur A, Rubens R, Khanna S, Gupta S (2019). Single-Dose Pharmacokinetics and Pharmacodynamics of IPX203 in Patients With Advanced Parkinson Disease: A Comparison With Immediate-Release Carbidopa-Levodopa and With Extended-Release Carbidopa-Levodopa Capsules. Clin Neuropharmacol.

[223] Giladi N, Caraco Y, Gurevich T, Djaldetti R, Adar L, Rachmilewitz Minei T, Oren S ND0612 (levodopa/carbidopa for subcutaneous infusion) achieves stable levodopa plasma levels when administered in low and high doses in patients with PD - MDS Abstracts. Mov Disord 32

[224] Poewe W, Stocchi F, Simuni T, Ellenbogen A, Leionen M, Rachmileqitz Mineu T, Case R, Kieburtz K, Olanow CW (2018) A multicenter, parallel-group, rater-blinded, randomized clinical study investigating the efficacy, safety and tolerability of 2 dosing regimens of ND0612

[225] Rosebraugh M, Kym P, Liu W, Facheris M, Benesh J (2019) A Novel Levodopa/Carbidopa Prodrug (ABBV-951) 24-Hour Continuous Subcutaneous Infusion Treatment for Parkinson’s Disease (P3.8-037). Neurology 92

[226] Pharmacokinetics of ABBV-951 Following 24-Hour Continuous Subcutaneous Infusion to Arm, Abdomen and Thigh - MDS Abstracts. In: MDS Abstracts. https://www.mdsabstracts.org/abstract/pharmacokinetics-of-abbv-951-following-24-hour-continuous-subcutaneous-infusion-to-arm-abdomen-and-thigh/. Accessed 20 Jun 2020

[227] Grosset D, European PD Therapy Compliance Study Group (2010). Therapy adherence issues in Parkinson’s disease. J Neurol Sci.

[228] Grosset D, Antonini A, Canesi M (2009). Adherence to antiparkinson medication in a multicenter European study. Mov Disord.

[229] LeWitt PA, Fahn S (2016). Levodopa therapy for Parkinson disease: A look backward and forward. Neurology.

[230] Nijhuis FAP, van den Heuvel L, Bloem BR, Post B, Meinders MJ (2019). The Patient’s Perspective on Shared Decision-Making in Advanced Parkinson's Disease: A Cross-Sectional Survey Study. Front Neurol.

